# Experimentally Validated Pharmacoinformatics Approach to Predict hERG Inhibition Potential of New Chemical Entities

**DOI:** 10.3389/fphar.2018.01035

**Published:** 2018-09-19

**Authors:** Saba Munawar, Monique J. Windley, Edwin G. Tse, Matthew H. Todd, Adam P. Hill, Jamie I. Vandenberg, Ishrat Jabeen

**Affiliations:** ^1^Research Center for Modeling and Simulation, National University of Science and Technology, Islamabad, Pakistan; ^2^Victor Chang Cardiac Research Institute, Sydney, NSW, Australia; ^3^School of Chemistry, The University of Sydney, Sydney, NSW, Australia

**Keywords:** hERG inhibitors, trosade de pointes, long QT syndrom, pharmcophore, GRIND, molecular docking, patch clamp

## Abstract

The hERG (human ether-a-go-go-related gene) encoded potassium ion (K^+^) channel plays a major role in cardiac repolarization. Drug-induced blockade of hERG has been a major cause of potentially lethal ventricular tachycardia termed Torsades de Pointes (TdPs). Therefore, we presented a pharmacoinformatics strategy using combined ligand and structure based models for the prediction of hERG inhibition potential (IC_50_) of new chemical entities (NCEs) during early stages of drug design and development. Integrated GRid-INdependent Descriptor (GRIND) models, and lipophilic efficiency (LipE), ligand efficiency (LE) guided template selection for the structure based pharmacophore models have been used for virtual screening and subsequent hERG activity (pIC_50_) prediction of identified hits. Finally selected two hits were experimentally evaluated for hERG inhibition potential (pIC_50_) using whole cell patch clamp assay. Overall, our results demonstrate a difference of less than ±1.6 log unit between experimentally determined and predicted hERG inhibition potential (IC_50_) of the selected hits. This revealed predictive ability and robustness of our models and could help in correctly rank the potency order (lower μM to higher nM range) against hERG.

## Introduction

The human ether-a-go-go-related gene (hERG) encoded potassium ion (K^+^) channel is an important component of the pore-forming α-subunits that conduct the rapidly activated delayed rectifier potassium current (*I*_*Kr*_) which is a major repolarization current of the cardiac action potential (Sanguinetti et al., 1995; Vandenberg et al., 2012).

A prolongation of the cardiac action potential and the QT interval on the surface electrocardiogram (ECG) has been associated with loss of function mutations in hERG (Yang et al., 2009; Sun et al., 2013; Zhang et al., 2013) or drug-trapping inside the central cavity of the hERG potassium channel and thus, may predispose to life-threatening ventricular tachyarrhythmia “Torsade-de-points” (TdP) (Roden, 2004; De Bruin et al., 2005). Other factors such as, electrolyte imbalance, ischemia are also reported (Sauer and Newton-Cheh, 2012). Several drugs including antibiotics (grepafloxacin Bischoff et al., 2000), antihistamine (astemizole Zhou et al., 1999), antimalarial (quinine Sǎnchez-Chapula et al., 2003, halofantrine Nosten et al., 1993), antipsychotic (sertindole, haloperidol, thioridazine, and pimozide Alvarez and Pahissa, 2010), and class III arrhythmia drugs (dofetilide Jurkiewicz and Sanguinetti, 1993 quinidine Roden et al., 1986) have been withdrawn from the market (De Ponti et al., 2002; Roden, 2004) due to drug induced QT prolongation. Furthermore, fifteen percent of drugs still on the market can cause QT prolongation and 4% are associated with Torsade-de-points (TdP) arrhythmia risk (data from www.crediblemeds.org). Moreover, it has been estimated that about 60% of drugs in development show hERG block. Therefore, there is considerable interest in screening for hERG block amongst future drug candidates (Redfern et al., 2003; Guth, 2007; Raschi et al., 2008).

**Graphical Abstract:**
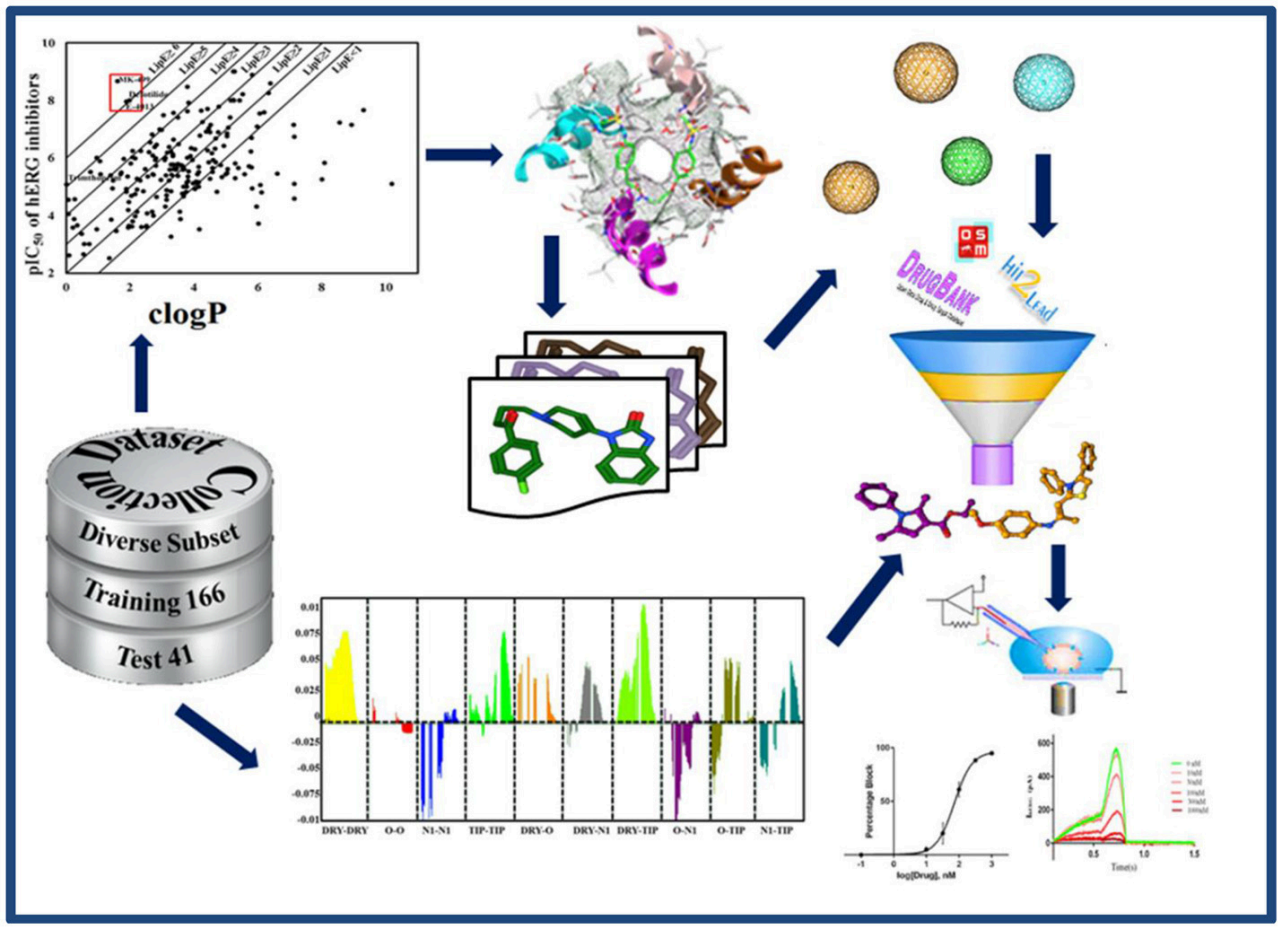
Integrated ligand and structure based pharmacoinformatic approach for the identification of hERG inhibition potential of New Chemical Entities (NCEs).

The International Conference on Harmonization (ICH), S7A (Food and Drug Administration, 2001) and S7B (Food and Drug Administration, 2005a) guidelines regarding technical requirements for registration of pharmaceuticals for human use requires preclinical assessment of QT prolongation risk prior to first administration in human. Although, hERG inhibition and the resulting risk of QT prolongation does not preclude clinical development, there are significant costs associated with this since any drugs that show hERG block must also be assessed clinically using a thorough QT/QTc study (Food and Drug Administration, 2005b) Furthermore, a compound without hERG liability is at a commercial advantage as compared to a competitor compound known to block the hERG channel.

According to the current guidelines on pre-clinical testing, the experimental hERG blockade ability of a compound is typically quantified in terms of concentration of the half-maximal inhibition (IC_50_) that is the most simple and amendable way (Redfern et al., 2003). However, kinetics of the channel, state dependent binding properties (Lee et al., 2016) and temperature dependent properties are also important factors in determining pro-arrhythmic risk assessment (Windley et al., 2018). The gold-standard method for the assessment of hERG liability is the patch-clamp electrophysiological assay (Hamill et al., 1981) on hERG transfected cells. However, this assay is costly, and not well-suited for extensive screening. Various other strategies including radiolabeled binding assays (Finlayson et al., 2001; Chiu et al., 2004), functional assays (Tang et al., 2001), and rubidium efflux assays (Chaudhary et al., 2006) have been developed but, these assays often result in significant under or overestimation of hERG potency when compared with electrophysiological methods (Hancox et al., 2008).

**Figure 1:**
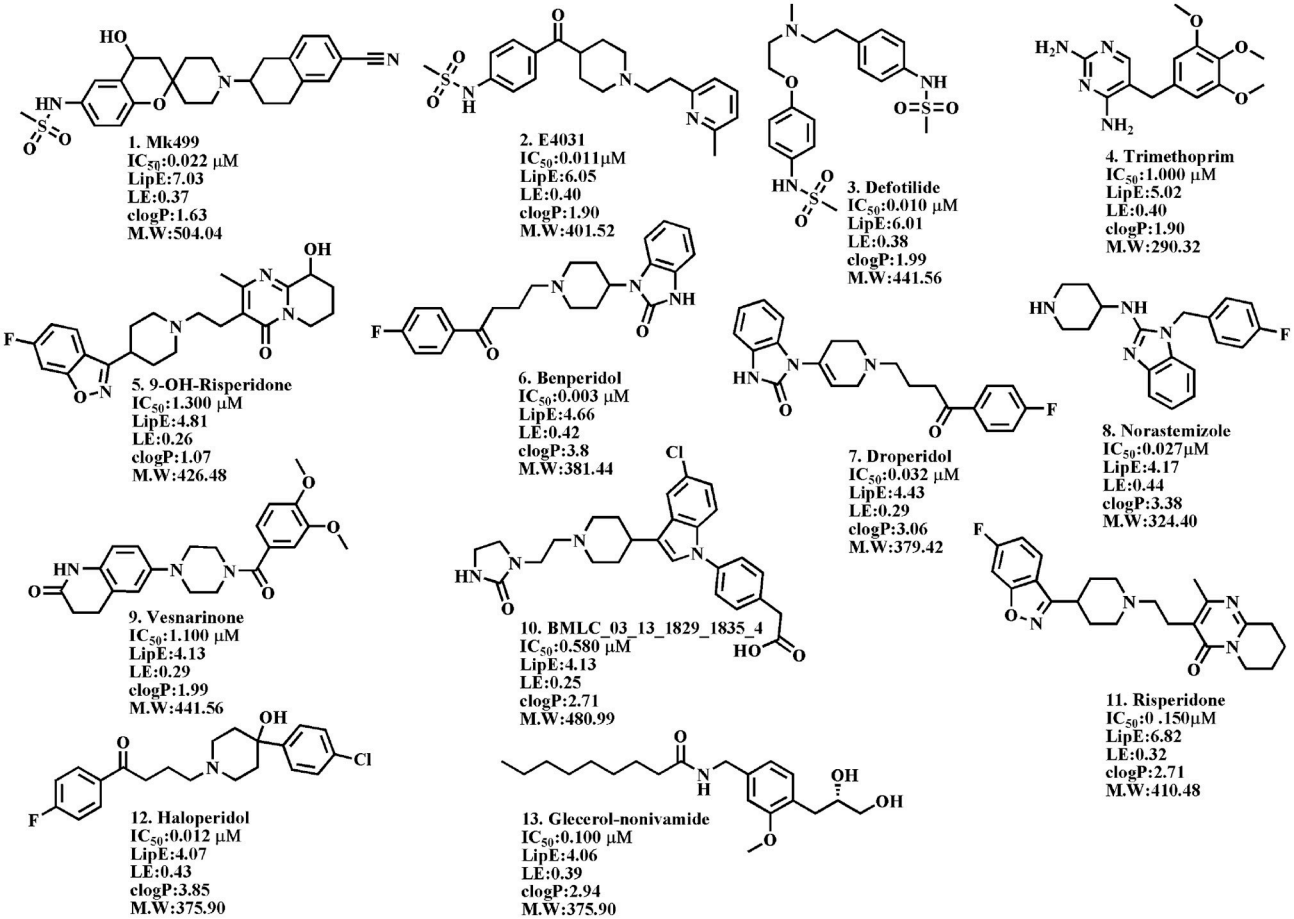
The hERG blockers with the best activity/molecular weight (ligand efficiency, LE) and activity/lipophilicity (Lipophilic efficiency, LipE) ratios.

To overcome these limitations, several *in silico* models including, classification or machine learning approaches (Dubus et al., 2006; Sun, 2006; Li et al., 2008, 2017; Thai and Ecker, 2008a; Kireeva et al., 2013; Liu et al., 2014; Chavan et al., 2016; Wang et al., 2016; Sun et al., 2017; Alves et al., 2018; Lu et al., 2018; Siramshetty et al., 2018; Wacker and Noskov, 2018), pharmacophore models (Cavalli et al., 2002; Aronov, 2005, 2006; Tan et al., 2012; Kratz et al., 2014; Wang et al., 2016; Chemi et al., 2017), 2D QSAR (Keserü, 2003; Bains et al., 2004; Seierstad and Agrafiotis, 2006; Song and Clark, 2006) and 3D QSAR (Ekins et al., 2002; Pearlstein et al., 2003) such as, comparative molecular similarity indices analysis (CoMSIA; Pearlstein et al., 2003), comparative molecular field analysis (CoMFA; Cavalli et al., 2002) and hologram QSAR (hQSAR; Keserü, 2003) have been developed to assess the pro-arrhythmia properties of drugs and their derivatives (Raschi et al., 2009). However, a generic 3D descriptors model on diverse data set of hERG inhibitors possessing good statistical parameters has not been reported yet. Here, we aim to develop a protocol using both ligand and structure based pharmacoinformatics approaches for the identification of general 3D structural features involved in hERG inhibition. Toward this goal, various GRid INdependent molecular Descriptor (GRIND) models have been developed using a diverse dataset of 207 hERG blockers extracted from the literature. Compounds with best activity/molecular weight (Hopkins et al., 2004) and activity/lipophilicity ratio (Leeson and Springthorpe, 2007; see Figure 1) were selected for building a pharmacophore hypothesis of hERG liability. Finally, proposed pharmacophore was validated by testing our identified hits using whole-cell patch clamp analysis on transfected hERG-CHO cells. Our model predictions show a difference of 1.6 and <0.1 log units from the experimentally determined pIC_50_ values of hits in the nanomolar (nM) to micromolar range (μM), respectively. Overall, our model can aid in the correct prediction of the hERG liability trends of NCEs from nM to μM range.

## Material and methods

### Dataset collection and refinement

A dataset of 1,428 compounds with known hERG inhibitory potency (IC_50_) values were collected from literature databases including, 938 compounds from the ChEMBL database (Bento et al., 2013), 140 compounds from the Fenichel database http://www.fenichel.net/pages/Professional/subpages/QT/Tables/pbydrug.htm) and 350 compounds from publications by Kramer et al. and Polak et al. (Kramer et al., 2008; Polak et al., 2009) as shown in Figure S1.

Inconsistencies in the dataset were removed by the application of various data refinement steps see Figure S2. Initially, all duplicates and fragments (molecular weight <200) in the data set were removed followed by manual correction of stereochemistry of stereoisomers. In order to remove any bias in the biological experiments, only those compounds for which hERG inhibitory potency (IC_50_) was determined using whole cell patch clamp technique were shortlisted. However, IC_50_ values of the selected compounds against hERG were calculated using two different mammalian expression system namely, Human Embryonic Kidney (HEK293) cell lines and the Chinese Hamster Ovary (CHO). Overall, a strong positive correlation *R*^2^ = 0.91 has been observed between reported pIC_50_ values of compounds tested using both HEK293 and CHO cell lines (Kramer et al., 2008; Polak et al., 2009; Figure S3). Therefore, compounds tested by whole cell patch clamp experiment using both HEK293 and CHO cell lines were kept in the final data set of 207 hERG inhibitors (see Supporting Information SMILES.csv file).

The 3D structures of all compounds in the dataset were generated using software MOE (I, 2013). Protonation and correction of partial charges were performed followed by energy minimization using MMFF94x force field (Halgren, 1996). Diverse subset selection procedure as reported by K.M. Thai et al. (Thai and Ecker, 2008b) was used to divide the data set into 80% training and 20% test set for further GRid-Independent Molecular Descriptor Analysis (GRIND).

### Grid-independent molecular descriptor (GRIND)

GRIND are alignment-free molecular descriptors, linked with 3D structural conformations of the dataset (Caron and Ermondi, 2007). Therefore, we used standard 3D conformations (Gasteiger et al., 1990), 3D energy minimized conformations (Gill et al., 1981), induced fit docking conformations (Sherman et al., 2006) in open state cryo-structure of hERG (Wang and MacKinnon, 2017), and molecular conformations obtained from stochastic search algorithm (Ferguson and Raber, 1989; see Supporting Information for conformational analysis details) to develop four different GRIND models. Briefly, molecular interaction fields (MIF) using four different probes including, “DRY” (hydrophobic), “N1” as (neutral flat amide: hydrogen bond donor hotspots), O (sp2 carbonyl oxygen: hydrogen bond acceptor) and TIP (molecular shape) were computed by software package Pentacle v 1.07 (Durán Alcaide, 2010). Total energy at each point was computed as the sum of Lennard-Jones (Elj), electrostatic (Eel), and hydrogen bond (Ehb) as described in Equation (1) by iteratively placing each probe at different GRID steps.

(1) $$\begin{array}{l} \text{Exyz}\;=\Sigma \text{Elj}+\Sigma \text{Eel}+\Sigma \text{Ehb} \end{array}$$

AMANDA algorithm (Durán et al., 2008) was applied for the extraction of the most relevant MIF using default energy cut-offs −0.5, −2.6, −4.2, −0.75 values for DRY, O, N1, and TIP probes, respectively. Consistently Large Auto And Cross Correlation (CLACC) algorithm was used to encode the pre-filtered nodes into four auto (DRY-DRY, O-O, N1-N1, TIP-TIP) and six cross (DRY-O, DRY-N1, DRY-TIP, O-N1, O-TIP, N1-TIP) correlograms (Durán Alcaide, 2010). These correlograms depict favorable/unfavorable interactions at Virtual Receptor Site (VRS). In order to reduce any redundancies in the large GRIND variable matrix, original variables are converted into default five principal components (PC) and latent variables (LV) for principal component analysis (PCA) and partial least square (PLS), respectively (Mannhold et al., 2006). Each independent GRIND model was built using Leave One Out (LOO) cross validation procedure of partial least square (PLS) analysis.

Overall, the statistical significance of the model was determined by *q*^2^ and *r*^2^ and standard deviation error prediction (SDEP). The *q*^2^ is the predictive ability of a model obtained by cross-validation procedure. Whereas, *r*^2^ is an index of model fitting on the training set defined as correlation coefficient of determinent. However, the standard deviation error of the predictions (SDEP) is an index of the model predictive ability obtained by cross-validation (Durán Alcaide, 2010).

In order to further probe the robustness of our final training model, two independent test sets were used for external validation. Test set I (41 compounds) was obtained after diverse subset selection procedure of the original dataset of know hERG inhibitors (Supporting Information SMILES.csv file) whereas, test set II (8 compounds) consists of an antimalarial Triazolopyrazine (TP) analogs possessing hERG liability as shown in Figure **4** taken from publically available data of Open Source Malaria (OSM) database (Williamson et al., 2016).

### Template selection for pharmacophore

In order to correlate VRS with 3D structural features of the hERG inhibitors, various pharmacophore models have been developed using selected templates with best activity/lipophilicity (LipE) and activity/molecular weight (LE) ratio (see details—in Supporting Information Methodology section and Figure S4).

Ligand-protein interaction guided pharmacophore models were developed using docked conformations of the selected templates (Figure 1) in open state cryo-structure (Wang and MacKinnon, 2017) and closed state homology model of hERG proposed by Stansfeld et al. (2007). The complete docking protocol and pose evaluation procedure have been discussed in Supporting Information (Figure S5).

### Pharmacophore modeling

The pharmacophoric sites and Gaussian radius size were used for optimizing the pharmacophoric hypothesis. Each pharmacophore model was tested against 207 compounds in our data set by setting an active threshold value of IC_50_ ≤ 40 μM. Quality of each pharmacophore was assessed by calculating Mathews correlation coefficient (MCC) as described in Equation (2).

(2) $$\begin{array}{l} \text{MCC}=\frac{TP*TN-FP*FN}{\sqrt{\left(TP+FP\right)\left(TP+FN\right)\left(TN+FP\right)\left(TN-FN\right)}} \end{array}$$

Overall, a final model with best statistical parameters was further used for virtual screening.

### Virtual screening

Finely selected pharmacophore model was used for virtual screening of the online ChemBridge database (Groom et al., 2016; http://www.chembridge.com/internal/) and Open Source Malaria (OSM) database (Williamson et al., 2016; https://ses.library.usyd.edu.au/handle/2123/15389). After pharmacophore screening 4,095 and 300 hits were obtained by Chembridge and OSM database, respectively. Various hit selection filters like Lipinski rule of five were applied to identify drug-like compounds (Lipinski et al., 2012), prediction of pIC_50_ values through GRIND model in order to consider potent compounds. The hERG inhibitory potency (pIC_50_) values of obtained hits were predicted by our final GRIND model using Pentacle software (Durán Alcaide, 2010). Finally, two compounds including one from ChemBridge (ID: 5931690) and one from OSM database (ID: OSM-S-31) with predicted IC_50_ values in nanomolar (nM) and micromolar (μM) range were selected for experimental evaluation by whole cell patch clamp technique. The NMR spectrum and associated data of both selected hits have been provided in Figures S7, S8.

## Biological section

### Whole cell patch clamp assay

#### Molecular biology/cell culture

Chinese hamster ovary (CHO) cells stably transfected Kv11.1 were purchased from American Type Culture Collection (ATTC reference PTA-6812). Whereas, the CHO cells were cultured in Hams F12 nutrient mix (ThermoFisher Scientific, Waltham, USA) containing 5% fetal bovine serum (Sigma-Aldrich, Sydney, Australia) and maintained at 37°C in 5% CO_2_. All chemicals were purchased from Sigma-Aldrich (Sydney, Australia) unless otherwise stated.

#### Electrophysiology

CHO cells were prepared 24 h before the experiment. The whole cell patch clamp electrophysiology studies were performed at 22°C. Glass capillary patch electrodes with resistance 2–4 MΩ were pulled with borosilicate glass using vertical two-stage puller (Harvard Apparatus, Holliston, USA). The pipettes were filled with internal solution containing (in μM):120 potassium gluconate, 20 KCl, 10 HEPES, 5 EGTA, 1.5 Mg-ATP and pH adjusted to 7.2 with KOH. The cells were superfused with external bath solution that contained (in μM): 130 NaCl, 12.5 glucose, 10 HEPES, 5 KCl, 1 MgCl_2_, 1 CaCl_2_, and pH was adjusted to 7.4 with NaOH. The calculated liquid junction potential for these solutions was −15 mV (Barry, 1994) and corrected by adjusting voltage pulse protocol for all experiments.

The whole-cell patch clamp mode was applied to cells in the voltage clamp configuration using Axopatch 200B amplifier (Molecular Devices, Sunny Vale, USA). Current signals were digitized at 5 kHz, filtered at 1 kHz and stored on IBM-compatible PC interfaced with a Digidata 1440A analog to digital converter (Molecular Devices, Sunny Vale, USA). Initial series resistance values were 2–5 MΩ which was compensated by at least 80% in all experiments. Leak subtraction was performed manually offline using acquisition software pClamp 10.2 (Molecular Devices, Sunny Vale, USA). A reusable microfluidic device (Dynaflow Resolve, Collectricon, Mölandal, Sweden) with <30 ms solution exchange time (Hill et al., 2014) was used for drug delivery. With the dynaflow system, different drug concentrations under laminar flow can be delivered to the cell.

##### Compounds solution

Both test compounds were prepared as a stock solution by dissolving in DMSO with a final concentration of 0.01% (v/v) in recording solution. The final concentration of DMSO if below 0.1% (v/v) has no effect on the activity of hERG channel (Walker et al., 1999).

##### Voltage protocol

To measure drug block “Step-Ramp” protocol was used (Crumb et al., 2016). Cells were depolarized from a holding potential to −80 to +20 mV for 500 ms to fully activate the channels. Cells were again repolarized to −80 mV over 250 ms.

##### Data analysis

For the analysis of drug block Clamp fit 10.2 software (Molecular Devices, Sunny Vale, USA) was used. Hill equation (Equation 3) was used for analyzing steady state dose response

(3) $$y=\frac{1}{1+{\left(\left[x\right]/IC_{50}\right)}^{n}H}$$

Where [x] represents the concentration of compound and ^n^**H** is the Hill coefficient (the slope parameter). The IC_50_ represents the concentration where there is a 50% blockage of channel current. Prism software was used for carrying out the statistical analysis.

## Result

**Figure 2:**
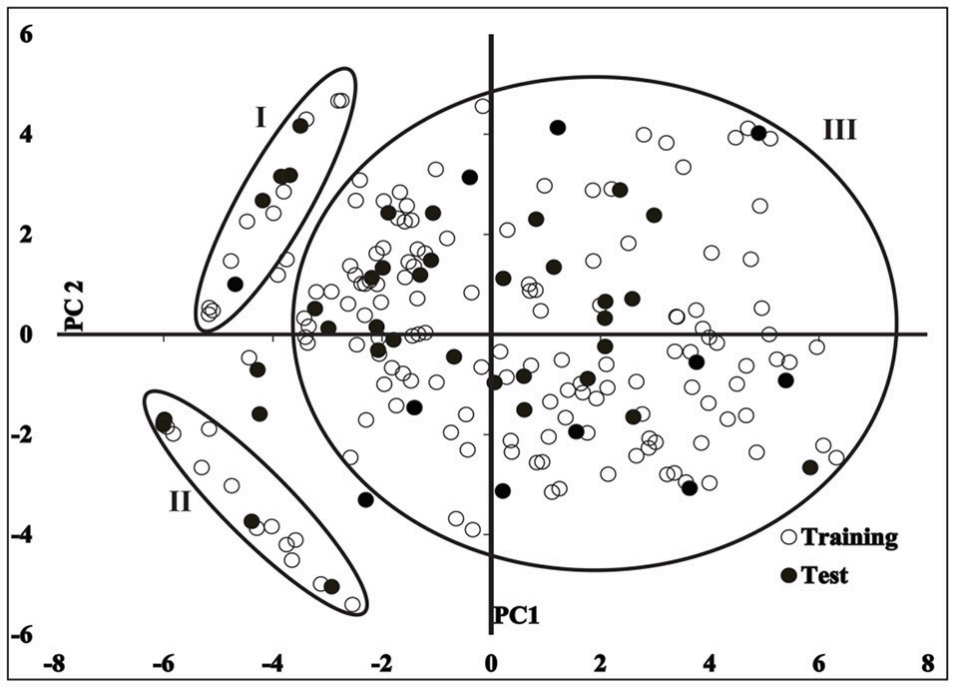
A plot between first two principal components (PC) illustrating descriptor space of 166 training set (hollow circles) and 41 test set (filled circles).

The structural variance of the training data was determined by principal component analysis (PCA; Wold et al., 1987) using complete sets of GRIND variables. Principal component scores of the training data vary from −5.8 to 6.2 as shown in Figure 2. Also projection of test set I and II reflect a principal component space. Briefly, principal component scores of first two principal components of GRIND variables explained only 37% of 3D structural variance of the dataset and divided it into three main clusters (I, II and III) as shown in Figure 2.

The chemical scaffolds of compounds in cluster I exhibit one hydrogen bond donor group that is complementary to O (carbonyl oxygen) probe at VRS that defines the most relevant regions for ligand-protein interactions. Compounds in cluster II exhibit one hydrogen bond acceptor and one hydrogen bond donor group in respective chemical scaffolds that depicts a complementary N1 (amide nitrogen) and O (carbonyl oxygen) probe, respectively at VRS. The remaining compounds in cluster III contain a maximum of three hydrogen bond acceptors and four hydrogen bond donor groups but otherwise cover a diverse range of chemical scaffolds. One representative compound from each cluster and the GRIND descriptor features of first two principal components are shown in Figure S10.

The importance of hydrogen bonding properties in hERG inhibition is already evident from various investigations (Aronov and Goldman, 2004; Choe et al., 2006; Farid et al., 2006; Chemi et al., 2017). However, the impact of the number of hydrogen bond acceptor and donor groups and their mutual distances in a given chemical scaffold on the hERG inhibition (IC_50_) has not been reported so far. Therefore, in the present investigation, the impact of number and mutual distances of hydrogen bond acceptor/donor groups on hERG inhibition potency of diverse chemical scaffolds has been illustrated with the help of the partial least square analysis (PLS) of GRid-INdependent molecular Descriptors (GRIND).

### Grid-independent molecular descriptor (GRIND) analysis

Partial least square (PLS) analysis using Leave One Out (LOO) cross-validation procedure (Elisseeff and Pontil, 2003) on individual set of molecular conformations of the data was performed to develop four independent GRIND models using the software package Pentacle v 1.07 (Durán Alcaide, 2010). However, only the GRIND model that was developed using standard 3D molecular conformational set of data showed statistically acceptable results with *q*^2^ of 0.54, *r*^2^ of 0.62 and Standard Deviation of Error Prediction (SDEP) of 0.94 (see Table 1). In order to further improve the statistical parameters and to remove the respective inconsistent GRIND variable, the fractional factorial design (FFD) algorithm (Baroni et al., 1993) was applied to each model as described by Pastor et al. (Pastor et al., 2000). Briefly, mutual comparison of statistical parameters of respective models after 1st and 2nd FFD cycles revealed a statistically significant final GRIND model with standard 3D molecular conformations of the data set after 2nd cycle of FFD as shown in Table 1.

**Table 1:** Statistical parameters of four different PLS models developed from GRIND using different 3D conformational inputs.

**Conformational method**	**Fractional factorial design (FFD) cycle**									**Comment FFD2 (LV2)**
	**Complete variable**			**FFD1**			**FFD2**			
	**${\text{q}}_{\text{LOO}}^{2}$**	***r*^2^**	**SDEP**	**${\text{q}}_{\text{LOO}}^{2}$**	***r*^2^**	**SDEP**	**${\text{q}}_{\text{LOO}}^{2}$**	***r*^2^**	**SDEP**	
Minimum energy conformation	0.38	0.51	1.09	0.45	0.56	1.09	0.45	0.56	1.09	Non-consistent with respect to auto and cross-correlogram
Stochastic search conformation	0.34	0.45	1.08	0.42	0.51	1.01	0.46	0.53	0.91	Non-consistent with respect to auto and cross-correlogram
Docking conformations	0.32	0.46	1.12	0.39	0.51	1.08	0.47	0.56	1.41	Non-consistent with respect to auto and cross-correlogram
Standard 3D conformations	0.54	0.62	0.94	0.61	0.67	0.86	**0.63**	**0.69**	**0.84**	Consistent with respect to TIP-TIP, DRY-TIP, and N1-N1 correlogram (Figure 6)

*The bold number represents finally selected model*.

**Figure 3:**
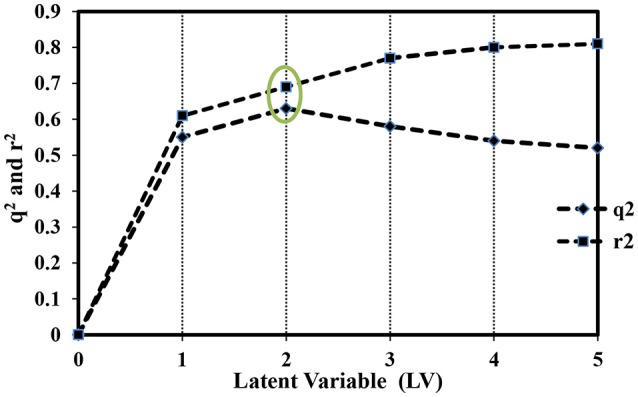
Plot representing the correlation between *q*^2^ and *r*^2^ values of the final GRIND model at different latent variables (LV-1-5).

Figure 3 shows a graph between *q*^2^ and *r*^2^ values of the final GRIND model up to the fifth latent variables (LV5). It illustrates a gradual increase in *r*^2^ values up to LV5. However, *q*^2^ values showed a decreasing trend after LV2. Therefore, a model with optimum statistics (*q*^2^ = 0.63, *r*^2^ = 0.69 and standard error of prediction (SDEP) = 0.84) was achieved at second latent variable (LV2), which was further used to correlate the structural variance of the data in general, and impact of number and mutual distances of hydrogen bond acceptors and donors in particular, with the hERG inhibition potential (pIC_50_).

**Figure 4:**
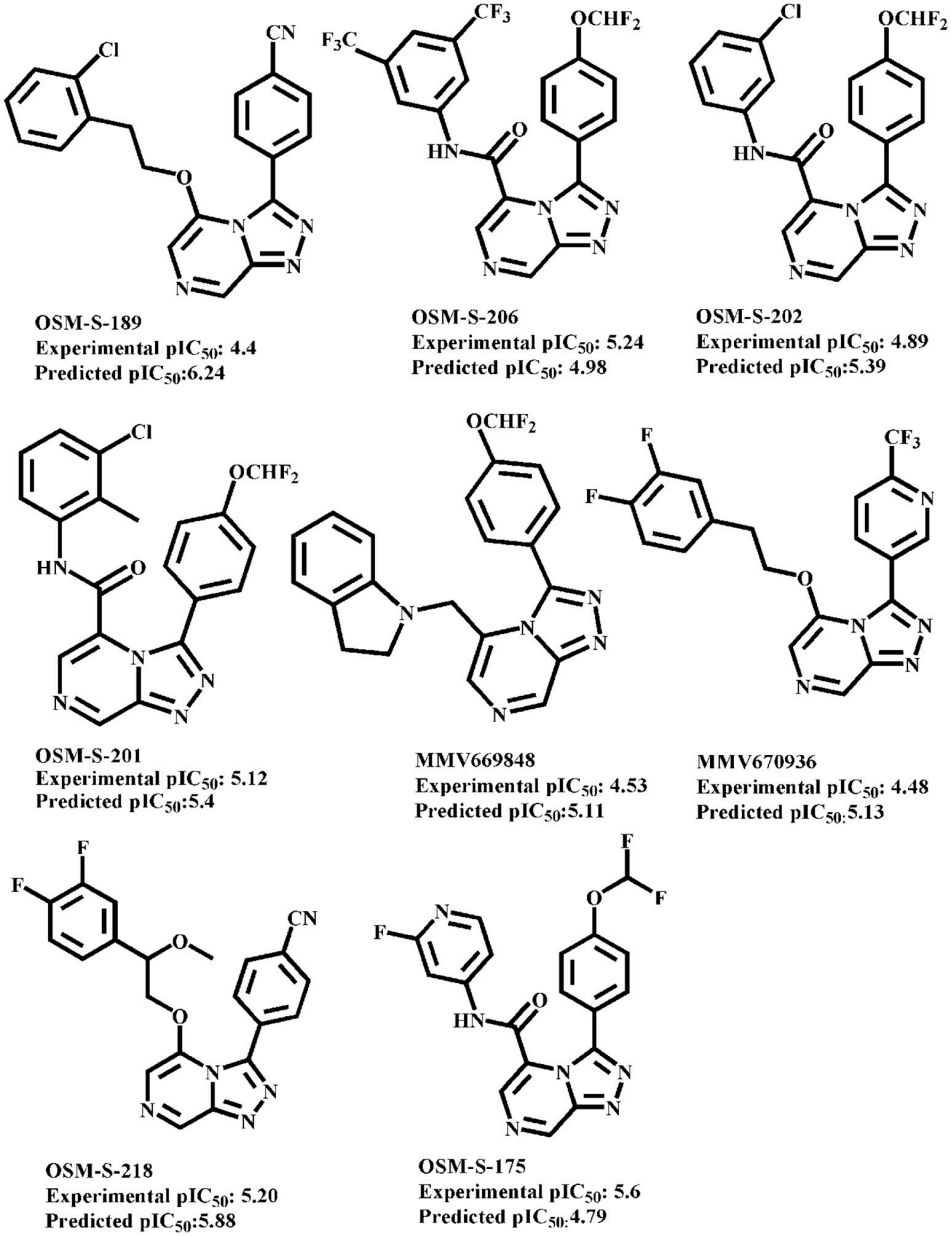
Experimental and predicted hERG inhibitory potential (pIC_50_) values of OSM database (test set II).

**Figure 5:**
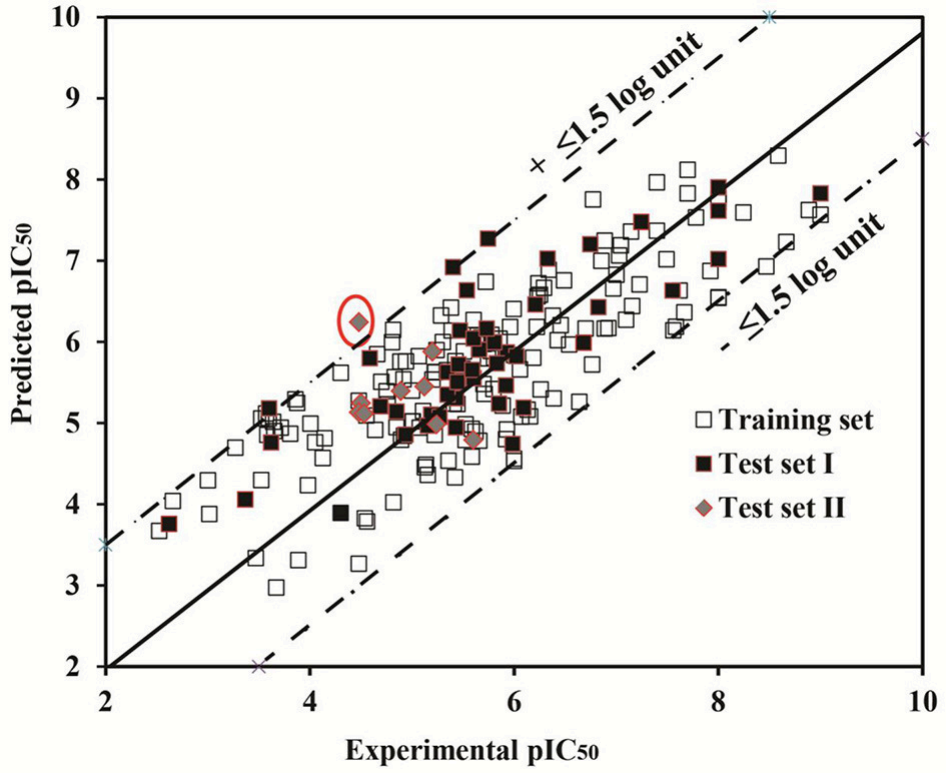
The plot of observed vs. predicted pIC_50_ values of the test set I (filled square), test set II (filled diamonds) projected on observed vs. predicted pIC_50_ values of the training set (hollow square).

The plot between experimental vs. predicted hERG inhibitory potency values (pIC_50_) of the training set and test set I (Information SMILES.csv file) and test set II (Figure 4) obtained after Leave One Out (LOO) cross-validation (Elisseeff and Pontil, 2003) and external validation procedure (Kiralj and Ferreira, 2009), respectively is shown in Figure 5. All compounds in training (*R*^2^: 0.67) as well as test set I (*R*^2^: 0.60) were predicted within a range of ±1.6 log units between experimental and predicted hERG inhibitory potency (pIC_50_) values. However, one compound of the test set II OSM-S-189 showed an outlier behavior with a difference of 1.8 log unit differences between experimental (pIC_50_: 4.4) predicted (pIC_50_: 6.2) hERG inhibition potential. Subsequently, the experimental protocol using whole cell patch clamp technique also delineates that our model may overestimate the affinity of drugs with experimentally determined pIC_50_ values in the high μM range.

**Figure 6:**
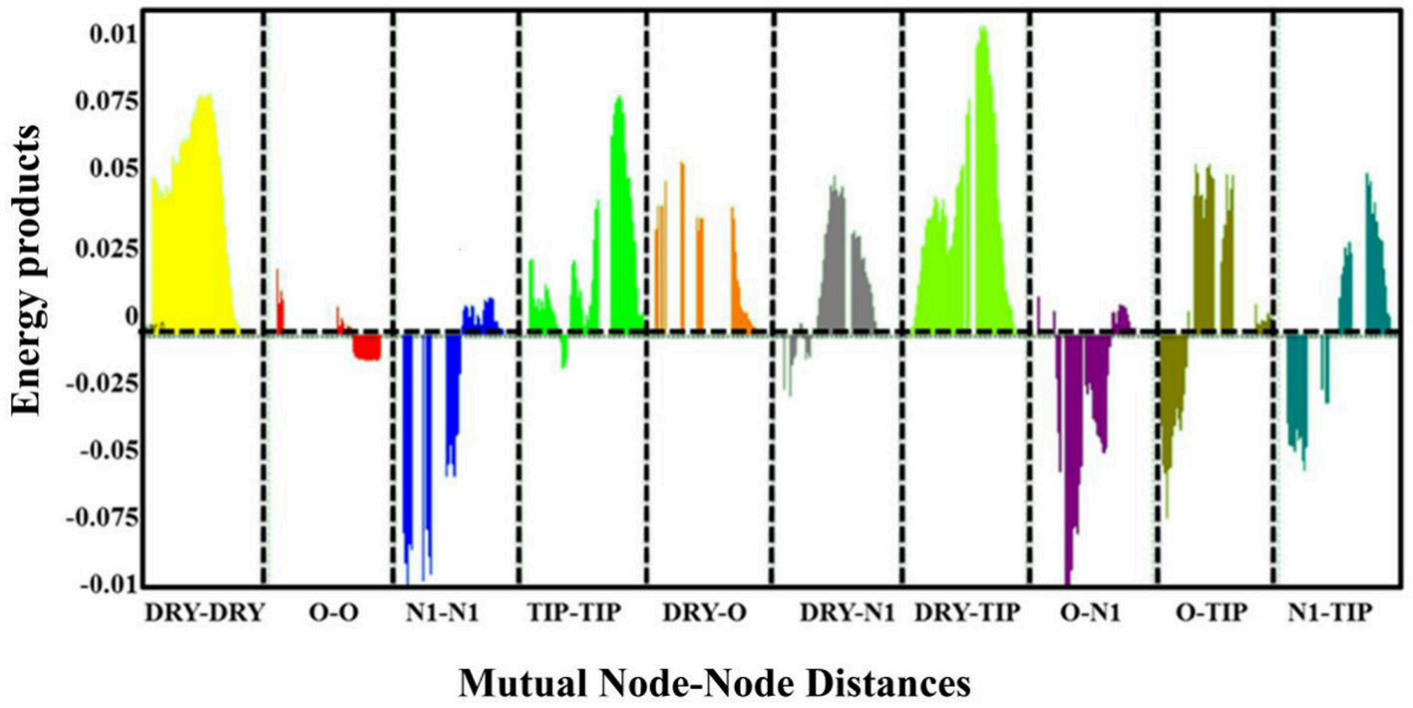
PLS co-efficient correlogram plot representing the GRIND variables exhibiting direct (positive values) and inverse(negative values) correlation with hERG inhibitory potency(pIC_50_) values.

The final GRIND model was used to probe 3D structural features of the data that might contribute to the drug trapping phenomena. Briefly, PLS coefficient profiles of auto and cross-correlograms in Figure 6 illustrates that DRY-DRY, TIP-TIP, DRY-TIP, and DRY-N1 pairs of variables map the 3D structural features of the data that play a major role in hERG inhibition potential (IC_50_). Whereas, O-N1 and N1-N1 variables depict the 3D structural features of the data that are more prominent in least potent hERG inhibitors (IC_50_: 214–3,000 μM).

**Figure 7:**
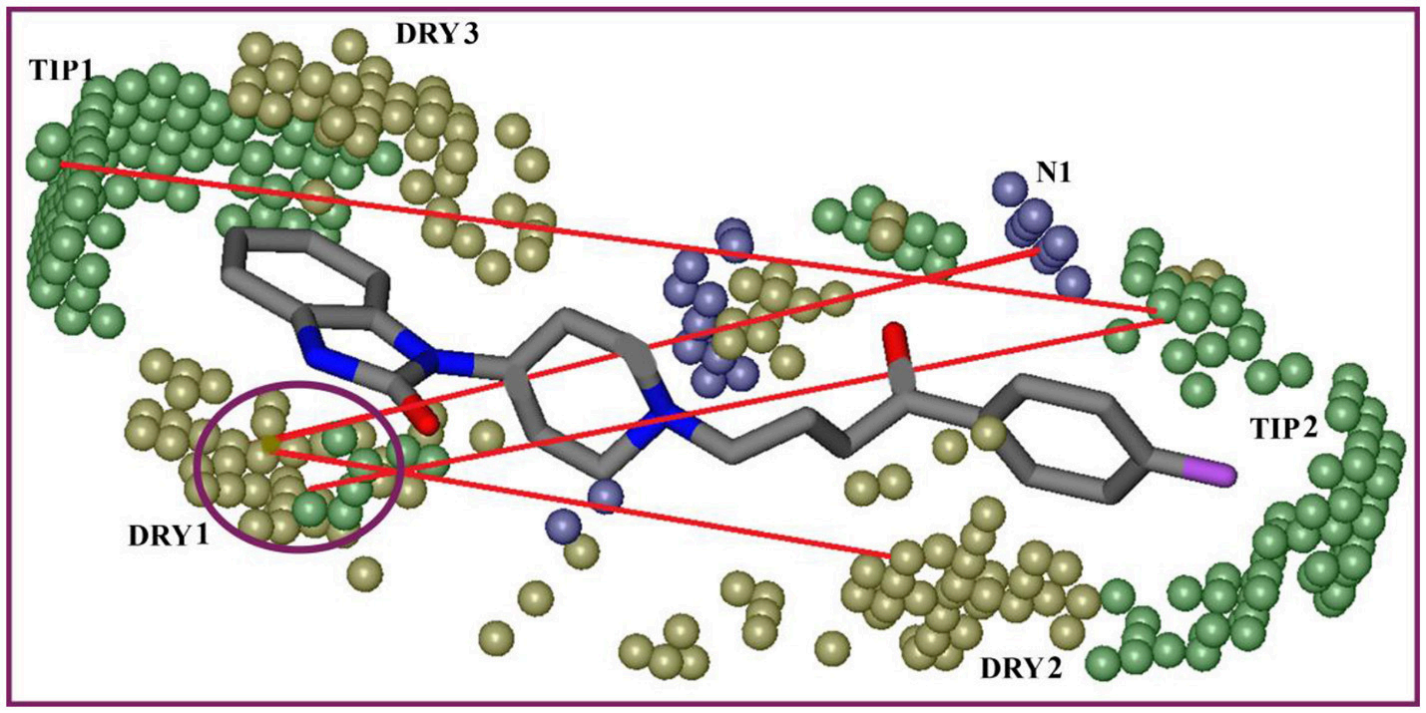
Shows the most relevant regions identified by GRIND model for ligand-hERG interaction. The contours define the virtual receptor site (VRS). DRY-DRY (yellow contours) representing the mutual distance of 14.0–14.4 A° between two hydrophobic molecular interaction fields (DRY1 and DRY2), TIP-TIP (green contours) feature showing a distance of 20.0–20.4 A° between two steric hotspots (TIP1 and TIP2), DRY-TIP representing a distance of 18.4–18.8 A° between hydrophobic molecular interaction field (DRY1: yellow) and steric hotspot (TIP2: green). DRY-N1 representing hydrophobic molecular interaction field (DRY1: yellow) at a distance of 10.8–11.8 A° from amide nitrogen representing hydrogen bond donor feature (N1: blue contours) that contribute positively to hERG blockage potential (pIC_50_). Interestingly, the molecular features mapped by DRY-DRY correlogram complement the molecular features translated by the highest peak of DRY**-**TIP cross-correlogram peaks shown in Figure 6. Both DRY-DRY and DRY-TIP auto and cross-correlograms corresponds to the hydrophobic moieties attached at both sides of the linker region.

The highest peak in **DRY-DRY** auto correlogram in Figure 6 delineates the presence of two hydrophobic regions at a distance of 14.0–14.4 A° in virtual receptor site of hERG inhibitors exhibiting IC_50_ values from 0.001 to 86 μM. In the present dataset of hERG inhibitors, this complements the distance between two or more aryl or aromatic moieties. However, these features are present at a shorter distance (5.6–6.0 A°) in virtual receptor site around least active hERG inhibitors. Similarly, the highest peak in **TIP-TIP** auto correlogram in Figure 6 represents the pair of steric hotspots that define the 3D molecular shape of the hERG blockers. It elucidates the presence of two steric hotspot regions at a mutual distance of 20.0–20.4 A° around hERG inhibitors with IC_50_ values ranges from 0.01 to 300 μM. Overall, both DRY-DR and TIP-TIP variables revealed the presence of polyaromatic rings on either side of the molecules as shown in Figure 7. It may point that hydrophobic molecular boundaries, perhaps owe to the unique shape of the hERG binding site as approximated by David et al. (Fernandez et al., 2004) by correlating the variation in hERG inhibition potential of various drugs with the change in van der Waals hydrophobic surface area of side chain residue Tyr_652 and Phe_656.

Similarly, DRY-N1 correlogram (Figure 6) maps the distance of a hydrophobic hotspot from a hydrogen bond donor hotspot region present at the VRS as shown in Figure 7. It has been observed that these two contours are present at a distance of 10.8–11.8 A° in VRS of highly potent hERG inhibitors with IC_50_ range from 0.023 to 0.74 μM whereas, in least active hERG blockers (IC_50_ > 100 μM) these two contours are present at 5.2–5.6 A° apart. Overall, our results show that one of the hydrophobic region (DRY1: yellow hotspots, enclosed by a circle Figure 7) might represent the most crucial contour as the distance of other pharmacophoric features including second hydrophobic region (DRY2), the steric molecular hotspot (TIP1) and a hydrogen bond donor (N1) contour has been calculated from this region. Thus, it is tempting to speculate that this hydrophobic region (DRY1) may provide an anchoring point for hydrophobic/aromatic interaction and the ligand may change its conformations in such a way to find complementary interaction points within binding site of hERG.

**Figure 8:**
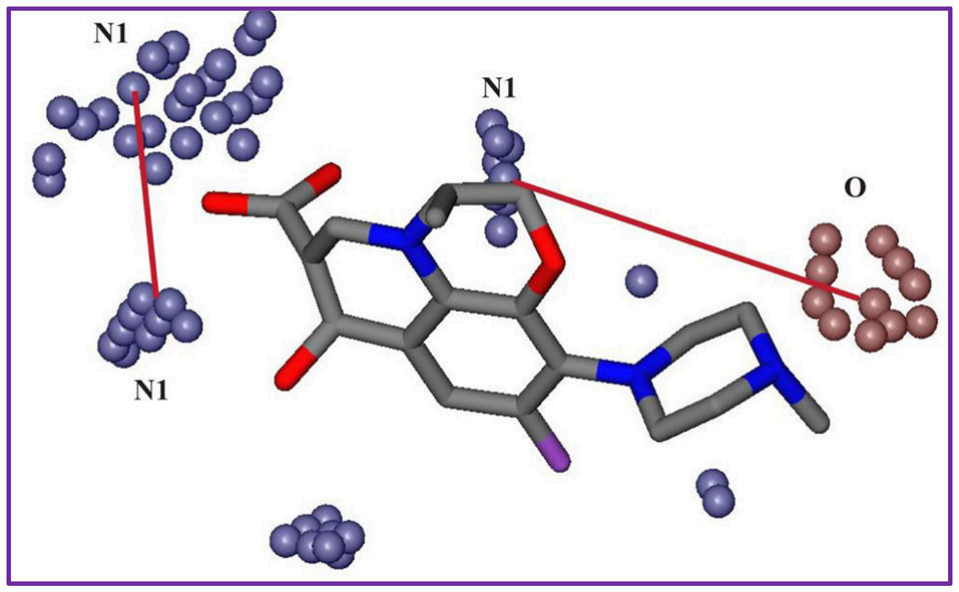
Shows 3D structural features of least active hERG inhibitors depicted by N1-N1 (blue contours) O-N1 (red and blue contours) PLS coefficient correlograms.

Interestingly, the highest negative peak in **N1-N1** correlogram in Figure 6 represents the variables that depict two hydrogen bond donor contours at a distance of 5.6–6.0 A° surrounding the least potent hERG blockers (IC_50_ values 252–2,200 μM). Similarly, the most negative correlogram for **O-N1** in Figure 6 illustrates a hydrogen bond acceptor and a hydrogen bond donor hotspot region, respectively at a mutual distance of 8.4–8.8 A° surrounding the data with hERG inhibition potential (IC_50_) value between 16 and 240 μM. Both the N1-N1 and O-N1 pair of probes have been identified surrounding 60% of compounds present in cluster III of PCA plot (Figure 2). These compounds exhibit 1–3 hydrogen bond acceptors and 1–4 hydrogen bond donor groups within diverse chemical scaffolds that complement respective N1-N1 and O-N1 hotspot regions as shown in Figure 8. However, none of the compounds in cluster I or cluster II of PCA plot (Figure 2) show a N1-N1 or O-N1 feature mainly due to the absence of second hydrogen bond donor and a hydrogen bond acceptor group within respective chemical scaffolds. Thus, it reflects that presence of two hydrogen bond acceptor groups in NCEs that complements N1-N1 hotspots of hydrogen bond donor probes at a distance of 5.6–6.0 A° may reduce the hERG inhibition potential. Also, one hydrogen bond donor group and one hydrogen bond acceptor group that complement to O-N1 contours at a distance of 8.4–8.8 A° might assist in reducing hERG liability. However, our results also emphasize that presence of one hydrogen bond acceptor group at a certain distance from a hydrophobic group within a chemical scaffold may increases the hERG liability (IC_50_). This is evident from hydrophobic (DRY: yellow) and hydrogen bond donor (N1: blue) contours (Figure 8) at a distance of 10.8–11.8 A° in virtual receptor space of all compounds in the present dataset of hERG inhibitors.

### Template selection for pharmacophore models

LipE profiles revealed that MK499, E4031, Dofetilide, and Trimethoprim showed LipE values of ≥5, logP between 0.4 and 3.0 and hERG inhibition potency (IC_50_) of 0.001–1 μM (Figure S4). This is in line with the already established thresholds of LipE and logP for the effectively transported drugs against therapeutic targets by Freeman-Cook et al. (Freeman-Cook et al., 2013). Therefore, MK499, E4031, Dofetilide, and Trimethoprim have been selected as templates for building pharmacophore based binding hypothesis of hERG.

While LipE normalizes for the lipophilic bias in potency description, ligand efficiency (LE) simply corrects for the size of a molecule by dividing the binding free energy of a compound by its heavy atom count. This approach is normally used in fragment-based drug design to select those fragments, which show optimal fit within the binding cavity of a protein/channel. Reynolds et al. (Reynolds et al., 2007) have shown that LE is generally biased toward smaller molecules. Therefore, the normalized size independent fit quality (FQ) score of the data set was assessed (as explained in Supporting Information Equation 5) MK499, E4031, Dofetilide, and Trimethoprim showed maximum FQ score (>1) which indicate optimal fit of these compounds within the bonding cavity of hERG channel (Figure S4). In order to remove any bias in the pharmacophore template selection criteria, here we also included those compounds exhibiting LipE > 4.0 and the LE > −0. 39 Kcal/mol/HA and FQ > 1 for pharmacophore query generation. These include 9-Hydroxy-risperidone, Benperidol, Droperidol, Norastemizole, Vesnarinone, BMCL-1835-4, Risperidone, Haloperidol, and Glycerol-nonivamide as shown in Figure 1. In order to probe most probable binding conformations of selected templates for building a pharmacophore, all 13 selected templates were docked into the recent cryo-EM structure of the hERG in its open state Wang and MacKinnon (Wang and MacKinnon, 2017). Additionally, the templates were also docked into closed conformational state of hERG homology model of Stansfled et al. (Stansfeld et al., 2007). The complete docking and pose evaluation protocol is discussed in Supporting Information Methods section.

Briefly, Table 2 provides an overview of the crucial amino acid residues interacting with selected templates in open and closed state of hERG and the GRIND contours that complement these interactions. However, a detail of the interaction pattern of selected templates has been provided in Supporting Information (Molecular Docking section, Figures S11–S15). Overall, final docking solutions of selected templates for the open state of hERG mainly occupy the basal cavity and show hydrophobic, π-cation and π-π interactions with amino acid residues Tyr_652, Phe_656, Ser_649 and Thr_623 in one or more subunits of hERG. Interestingly, these interactions complement the DRY-DRY, DRY-TIP and DRY-N1 probes representing the hydrophobic virtual receptor space (Figure 7) depicted by PLS coefficient correlogram in Figure 6. Whereas, in the closed state of the channel, the binding positions of the templates are shifted near the bottom of the selectivity filter and carbonyl oxygen of (MK499, Dofetilide, Trimethoprim etc.) form hydrogen bonding with Ser_624, as shown in Figures S11–S15. Additionally, π-π interactions between Phe_656/Tyr_652 and aromatic/hydrophobic moieties of templates have been identified (Figures S11–S15). Overall, these interactions correspond to GRIND mapped distance between a hydrophobic group and a hydrogen bond acceptor group within the highly potent hERG inhibitors as depicted by DRY-N1 correlogram in Figure 6. The interaction of hydrophobic substitutions with one of the four concentric rings of Tyr_652 and Phe_656 in the basal cavity and with Ser_649 and Thr_623 at the bottom of the pore helix has already been reported for drug trapping within hERG (Lees-Miller et al., 2000; Mitcheson et al., 2000; Fernandez et al., 2004). Specifically, the identified binding profiles of **E4031**, **MK499, dofetilide and 9-Hydroxy Risperidone** (Figures S11–S13) are also supported by previously reported interactions of these drugs in open and closed conformational state of hERG (Mitcheson et al., 2000; Dempsey et al., 2014) Thus, these further qualify the probable binding solutions of templates in open and closed conformational state of the hERG for pharmacophore modeling and virtual screening.

**Table 2:** Showing importing interacting residues of selected templates in open and closed conformation and their complementary GRIND features.

**Sr No**	**Template**	**Interacting residues in recent Cryo_EM structure in open conformation state of hERG**	**Complementary GRIND contours**	**Interacting residues in close conformation state of hERG**	**Complementary GRIND contours**
1	MK-499	Met_645, Gly_648, Leu_622	DRY-N1, DRY-N1	Tyr_652, Phe_656	DRY-DRY TIP-TIP
2	E4031	Ser_624, Ser_649, Lue_622	DRY-O Dry-N1	Ser_624, Phe_656	DRY-N1 DRY-DRY
3	Dofetilide	Met_645, Tyr_652, Thr_623, Ser_649	DRY-DRY DRY-N1	Ser624, Ser649	DRY-N1
4	Trimethoprim	Ser_624, Thr_623	DRY-N1	Tyr_623, Ser_624	DRY-N1
5	9OH-Risperidone	Tyr_652, Ser_624	DRY-DRY DRY-N1	Ser_649, Tyr_652	DRY-DRY DRY-N1
6	Benperidol	Ser_621, Tyr_652	DRY-N1 DRY-DRY	Ser_649, Tyr_652 Phe_656	DRY-N1 DRY-DRY DRY-TIP
7	Droperidol	Tyr_652, Ser_624	DRY-DRY DRY-N1	Ser_624, Ser_649	DRY-N1
8	Norastemizole	Ser_624, Tyr_652	DRY-O DRY-DRY	Ser_624, Lue_622 Gly_648	DRY-N1 DRY-O
9	Vesnarinone	Leu_622, Tyr_652	DRY-O DRY-DRY	Phe_656, Ser_649	DRY-DRY DRY-N1
10	BMLC_1835_4	Ser_621, Tyr_652	DRY-N1 DRY-TIP	Leu_622, Thr_623	DRY-N1
11	Risperidone	Ser_624, Tyr_652	DRY-N1 DRY-DRY	Ser_649	DRY-N1
12	Haloperidol	Phe_624, Tyr_652	DRY-DRY DRY-N1	Ser_624, Phe_656	DRY-N1 DRY-DRY
13	Glycerol-nonivamide	Tyr_652, Ser_624	DRY-N1, DRY-DRY	Ser_649	DRY-N1

### Pharmacophore-based virtual screening (VS)

**Figure 9:**
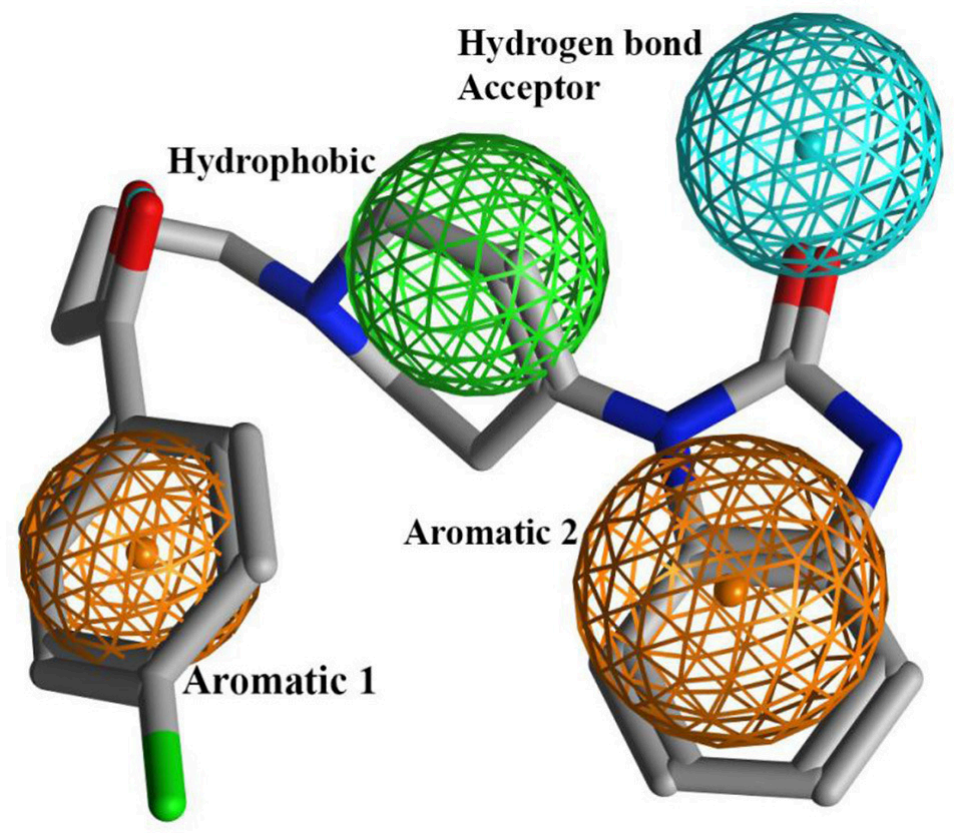
Showing statistically significant hERG inhibition pharmacophore model obtained using Droperidol docked in the closed conformational state of hERG as template. The pharmacophore consists of two aromatic, one hydrophobic and one hydrogen bond acceptor feature.

Finally selected binding solutions of 13 different templates (Figure 1) in open as well as closed conformations of hERG were used to build respective interaction profiles guided hERG inhibition pharmacophores. Prior to validation these 13 compounds were excluded from the whole dataset. Two statistically significant (maximum true positive and true negative) models per template in open and closed states of hERG have been selected for further feature analysis. 24 out of 26 pharmacophore models exhibit two aromatic, one hydrophobic and one hydrogen bond acceptor features at variable mutual distances. However, one hydrophobic feature was absent in two of the models built using Haloperidol template as shown in Table 3. Overall, only a slight difference in mutual distances of pharmacophore features in all 26 models has been observed which mainly reflect the dynamic nature of the complex physiological system. Additionally, all 26 pharmacophore models showed similar model statistics Table 3. However, a final pharmacophore with optimal true positive (TP: 71%), true negative (TN: 75%), sensitivity (0.72) and specificity rate (0.75) and Mathews correlation coefficient (MCC: 0.72) as shown in Table 3 and Figure 9 was selected for further comparison with GRIND features and virtual screening of validation set.

**Table 3:** Statistical parameters and mutual pharmacophoric features distances (A°) of pharmacophore models developed using most probable binding conformations of selected templates in open and closed state of hERG.

**Compound**		**hERG open state conformation model**							**hERG close state conformation model**						
		**Model template**	**Model distances A°**					**Model statistics**	**Model template**	**Model distances A°**					**Model statistics**
1	MK-499	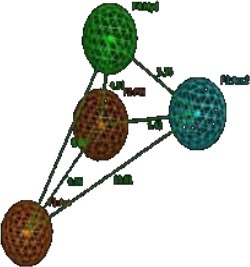		Aro 1	Hyd	Aro 2	HBA	TP: 102/177 TN:75/177 FP:7/32 FN:25/32 MCC = 0.70	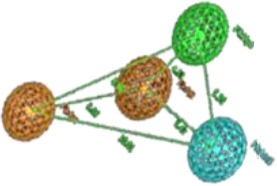		Aro1	Hyd	Aro.2	HBA	TP: 108/177 TN:69/177 FP:6/32 FN:24/32 MCC = 0.71
			Aro1	0	5.8	6.6	6.1			Aro1	0	5.3	6.1	5.8	
			Hyd	5.8	0	7.7	6.3			Hyd	5.3	0	4.7	6.3	
			Aro2	6.6	7.7	0	8.2			Aro2	6.1	4.7	0	7.2	
			HBA	6.1	6.3	8.2	0			HBA	5.8	6.3	7.2	0	
2	E4031	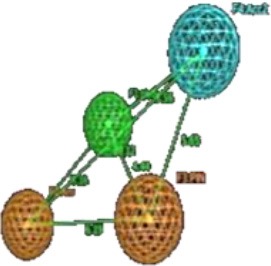		Aro 1	Hyd	Aro.2	HBA	TP: 105/177 TN:72/177 FP:9/32 FN:23/32 MCC = 0.69	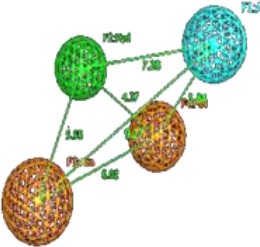		Aro1	Hyd	Aro.2	HBA	TP: 105/177 TN:72/177 FP:9/32 FN:23/32 MCC = 0.69
			Aro1	0	7.2	5.1	8.1			Aro1	0	6.3	5.4	6.8	
			Hyd	7.2	0	6.2	5.6			Hyd	6.3	0	5.7	4.3	
			Aro2	5.1	6.2	0	6.1			Aro2	5.4	5.7	0	5.5	
			HBA	8.1	5.6	6.1	0			HBA	6.8	4.3	5.5	0	
3	Dofetilide	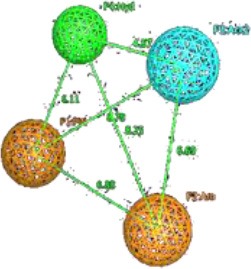		Aro 1	Hyd.1	Aro.2	HBA	TP:108/177 TN:71/177 FP:8/32 FN:24/32 MCC = 0.69	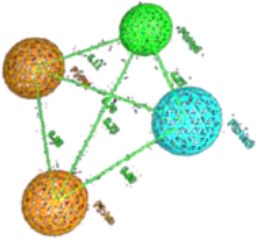		Aro 1	Hyd	Aro.2	HBA	TP:110/177 TN:73/177 FP:6/32 FN:26/32 MCC = 0.70
			Aro1	0	6.3	6.6	7.3			Aro1	0	8.2	5.1	6.6	
			Hyd	6.3	0	6.4	5.2			Hyd	8.2	0	4.6	6.8	
			Aro2	6.6	6.4	0	8.2			Aro2	5.1	4.6	0	6.1	
			HBA	7.3	5.2	8.2	0			HBA	6.6	6.8	6.1	0	
4	Trimethoprim	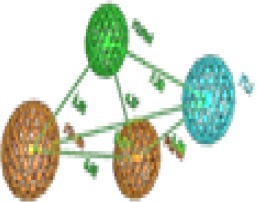		Aro 1	Hyd	Aro.2	HBA	TP: 99/177 TN:78/177 FP:9/32 FN:23/32 MCC = 0.69	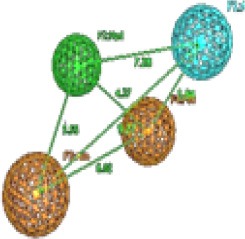		Aro 1	Hyd	Aro.2	HBA	TP:105/177 TN:72/177 FP:7/32 FN: 25/32 MCC = 0.69
			Aro1	0	4.3	5.6	5.3			Aro1	0	4.6	5.1	4.3	
			Hyd	4.3	0	4.4	5.8			Hyd	4.6	0	3.8	5.2	
			Aro2	5.6	4.4	0	4.4			Aro2	5.1	3.8	0	5.1	
			HBA	5.3	5.8	4.4	0			HBA	4.3	5.2	5.1	0	
5	9-Hydroxy -Risperidone	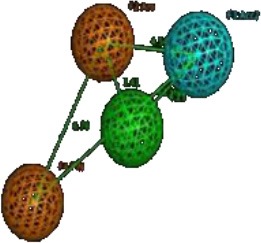		Aro 1	Hyd	Aro.2	HBA	TP:112/177 TN:66/177 FN:20/32 FP:12/32 MCC = 0.69	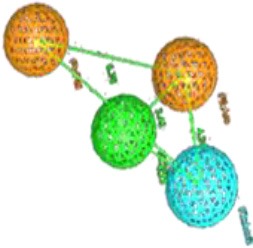		Aro 1	Hyd	Aro.2	HBA	TP:123/177 TN:54/177 FN:25/32 FP:7/32 MCC = 0.69
			Aro1	0	4.3	6.1	4.4			Aro1	0	3.6	5.1	4.3	
			Hyd	4.3	0	4.1	5.2			Hyd	3.6	0	4.2	5.2	
			Aro2	6.1	4.1	0	4.4			Aro2	5.1	4.2	0	7.1	
			HBA	4.4	5.2	4.4	0			HBA	4.3	5.2	7.1	0	
6	Benperidol	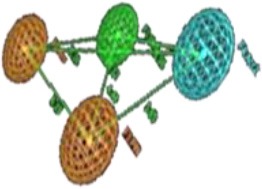		Aro 1	Hyd	Aro.2	HBA	TP:120/177 TN:57/177 FP:12/32 FN:20/32 MCC = 0.66	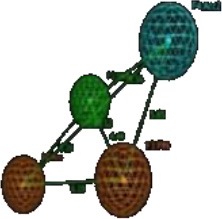		Aro 1	Hyd	Aro.2	HBA	TP:122/177 TN:55/177 FP:10/32 FN:22/32 MCC = 0.66
			Aro1	0	4.6	5.1	7.4			Aro1	0	6.2	5.4	6.8	
			Hyd	4.6	0	5.5	4.8			Hyd	6.2	0	4.7	6.2	
			Aro2	5.1	5.5	0	4.1			Aro2	5.4	4.7	0	4.8	
			HBA	7.4	4.8	4.1	0			HBA	6.8	6.2	4.8	0	
7	Droperidol	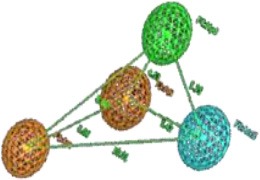		Aro 1	Hyd	Aro.2	HBA	TP:119/177 TN:68/177 FP:6/32 FN:26/32 MCC = 0.70	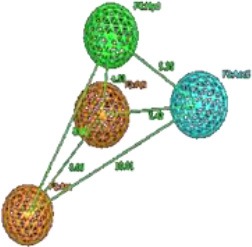		**Aro 1**	**Hyd**	**Aro.2**	**HBA**	**TP:119/177** **TN:68/177** **FN:24/32** **FP:6/32** **MCC** = **0.72**
			Aro1	0	5.3	4.2	4.1			**Aro1**	**0**	**4.4**	**5.6**	**5.8**	
			Hyd	5.3	0	5.7	5.8			**Hyd**	**4.4**	**0**	**6.3**	**4.7**	
			Aro2	4.2	5.7	0	6.1			**Aro2**	**5.6**	**6.3**	**0**	**6.0**	
			HBA	4.1	5.8	6.1	0			**HBA**	**5.8**	**4.7**	**6.0**	**0**	
8	Nor-astemizole	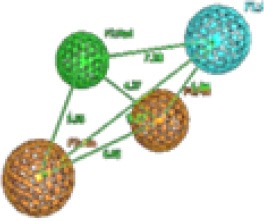		Aro 1	Hyd	Aro.2	HBA	TP:119/117 FN:26/32 FP:6/32 TN:68/117 MCC = 0.70	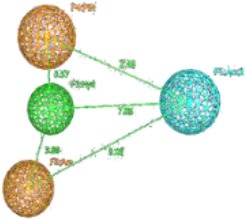		Aro 1	Hyd	Aro.2	HBA	TP:121/177 TN:56/177 FN:25/32 FP:7/32 MCC = 0.67
			Aro1	0	5.5	5.2	6.4			Aro1	0	3.8	5.8	9.0	
			Hyd	5.5	0	5.1	3.9			Hyd	3.8	0	3.5	7.0	
			Aro2	5.2	5.1	0	5.8			Aro2	5.8	3.5	0	7.4	
			HBA	6.4	3.9	5.8	0			HBA	9.0	7.0	7.4	0	
9	Vesnarinone	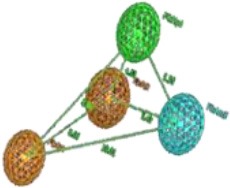		Aro 1	Hyd	Aro.2	HBA	TP:109/177 TN:68/177 FP:10/32 FN:22/32 MCC = 0.68	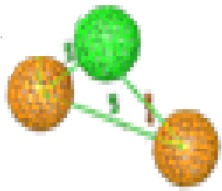		Aro 1	Hyd	Aro.2	HBA	TP:129/177 TN:48/177 FN:8/32 TN:24/32 MCC = 0.65
			Aro1	0	3.8	4.5	4.4			Aro1	0	9.4	6.0	NA	
			Hyd	3.8	0	5.3	5.5			Hyd	9.4	0	4.6	NA	
			Aro2	4.5	5.3	0	7.2			Aro2	6.0	4.6	0	NA	
			HBA	4.4	5.5	7.2	0			HBA	NA	NA	NA	0	
10	BMLC_1835_4	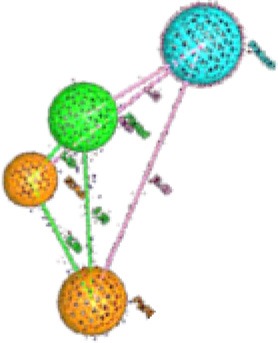		Aro 1	Hyd	Aro.2	HBA	TP:120/177 TN:57/177 FP:24/32 FN:8/32 MCC = 0.67	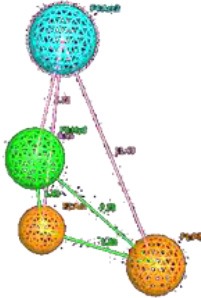		Aro 1	Hyd	Aro.2	HBA	TP:123/177 TN:54/177 FP:25/32 FN:7/32 MCC = 0.66
			Aro1	0	4.1	6.5	5.7			Aro1	0	3.4	5.8	3.4	
			Hyd	4.1	0	5.3	5.5			Hyd	3.4	0	7.5	5.7	
			Aro2	6.5	5.3	0	11.5			Aro2	5.8	7.5	0	11.9	
			HBA	5.7	5.5	11.5	0			HBA	3.4	5.7	11.9	0	
11	Resperidone	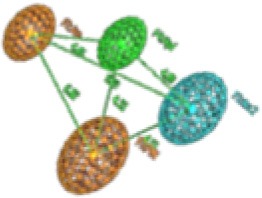		Aro 1	Hyd.	Aro.2	HBA	TP:117/177 TN:60/177 FP:8/32 FN:24/32 MCC = 0.68	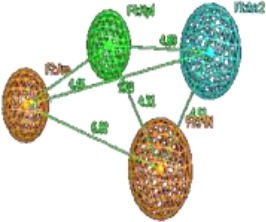		Aro 1	Hyd	Aro.2	HBA	TP:120/177 FP:24/32 TN:57/177 FN:8/32 MCC = 0.67
			Aro1	0	5.1	6.7	8.7			Aro1	0	4.4	6.8	9.0	
			Hyd	5.1	0	5.3	4.5			Hyd	4.4	0	4.3	4.8	
			Aro2	6.7	5.3	0	5.6			Aro2	6.88	4.3	0	4.5	
			HBA	8.7	4.5	5.6	0			HBA	9.0	4.8	4.5	0	
12	Haloperidol	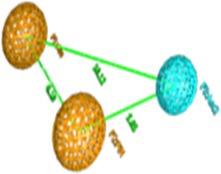		Aro 1	Hyd	Aro.2	HBA	TP:130/177 TN:47/177 FP:24/32 FN:8/32 MCC = 0.64	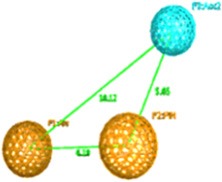		Aro 1	Hyd	Aro.2	HBA	TP:132/177 TN:45/177 FP:24/32 FN:8/32 MCC = 0.66
			Aro1	0	NA	4.8	6.7			Aro1	0	NA	10.1	6.1	
			Hyd	NA	0	NA	NA			Hyd	NA	0	NA	NA	
			Aro2	4.8	NA	0	8.3			Aro2	6.1	NA	0	5.6	
			HBA	6.7	NA	8.3	0			HBA	10.1	NA	5.6	0	
13	Glycerol -Nonivamide	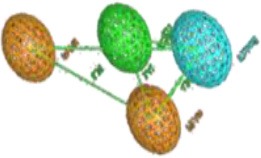		Aro 1	Hyd	Aro.2	HBA	TP:120/177 TN:57/177 FP:23/32 FN:9/32 MCC = 0.67	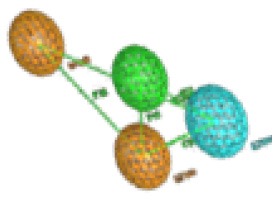		Aro 1	Hyd	Aro.2	HBA	TP:122/177 TN:55/177 FP:22/32 FN:10/32 MCC = 0.66
			Aro1	0	3.4	4.7	3.1			Aro1	0	3.7	5.2	3.1	
			Hyd	3.4	0	4.4	4.5			Hyd	3.7	0	5.3	4.1	
			Aro2	4.7	4.4	0	8.1			Aro2	5.2	5.3	0	7.6	
			HBA	3.1	4.5	8.1	0			HBA	3.1	4.1	7.6	0	

*The bold values represents finally selected model*.

### Experimental validation

**Figure 10:**
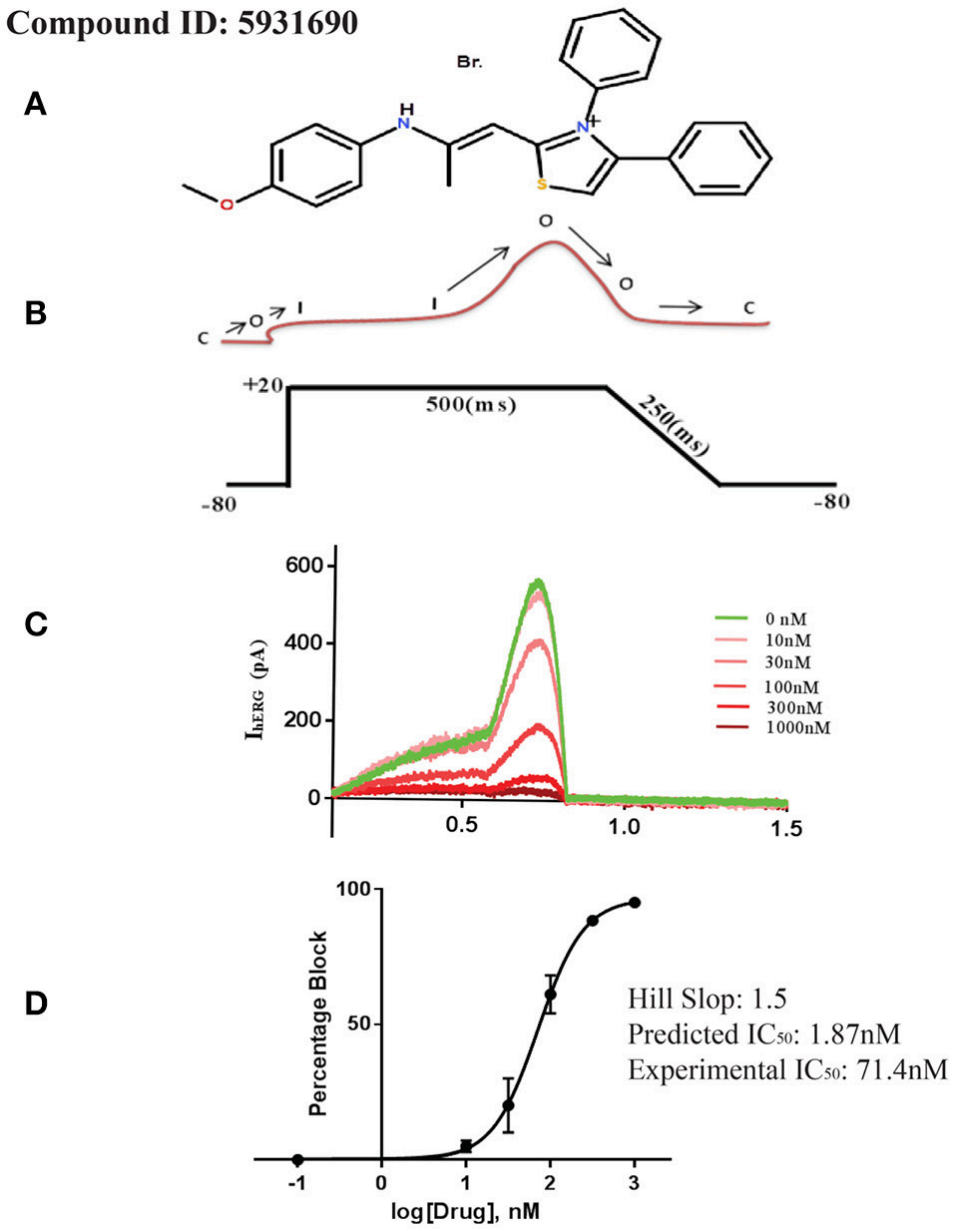
**(A)** 2D structure of selected compound from ChemBridge database. **(B)** Step ramp protocol. **(C)** Plot of current (pA) traces vs. time(s): Green sweep is showing maximum current passing through the channel when no drug is applied to the channel. The gradual decrease in current peak is showing channel inhibition in response to various dose concentrations. **(D)** Dose-response curve showing percentage blockade of hERG current against various drug concentration.

**Figure 11:**
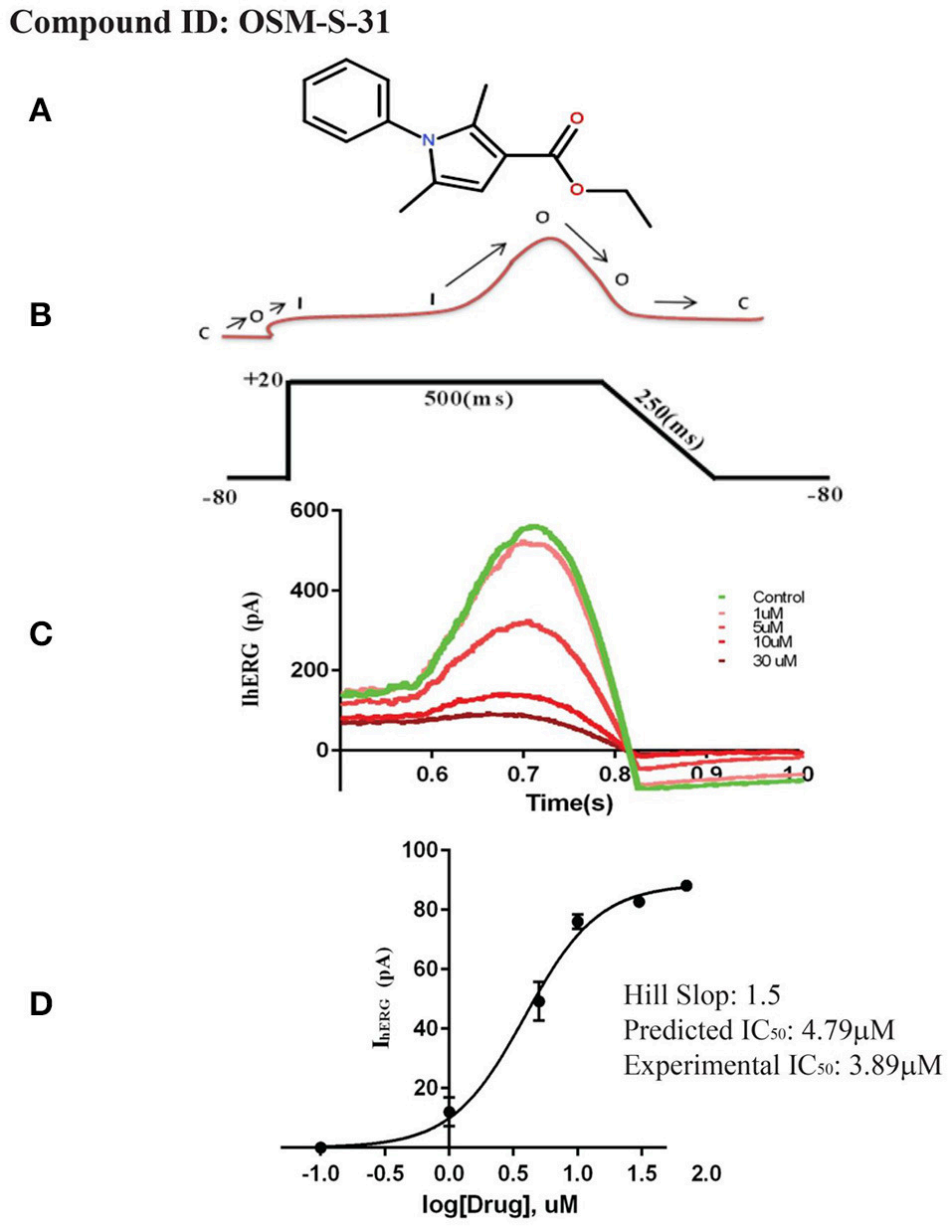
**(A)** 2D structure of selected compound from OSM database. **(B)** Step ramp protocol. **(C)** Plot of current (pA) traces vs. time (s): Green sweep is showing maximum current passing through the channel when no drug is applied to the channel. The gradual decrease in current peak is showing channel inhibition in response to various dose concentrations. **(D)** Dose-response curve showing percentage blockade of hERG current against various drug concentration.

To experimentally test our identified pharmacophore features, a data set of 458,921 compounds from ChemBridge data base (Groom et al., 2016) and 405 compounds from OSM database (Williamson et al., 2016) have been screened against the final hERG inhibition model. Overall, 4,905 compounds from ChemBridge database (Groom et al., 2016) and 300 compounds from OSM (Williamson et al., 2016) screening set have been identified as hits. Various hit selection filters as described in Methods section were applied to further prioritize the hits that result in 340 and 170 hits from ChemBridge (Groom et al., 2016) and OSM database (Williamson et al., 2016), respectively. In order to further reduce the number of hits, hERG inhibition potential of final hit structures from both ChemBridge and OSM databases has been predicted using our final GRIND model (data not shown). The applicability domain of 100 top predicted hits from each of the data sets was further evaluated by principle components (PC) scores. The PC scores of 1st two principal components ranges from −5.0 to 5.0 fitted within the applicability domain of the training data (−5.8 to 6.2). Finally, one compound from each database with highest predicted hERG inhibition potential (IC_50_) was applied to hERG-CHO cells and pIC_50_ determined using the whole cell patch clamp technique. These include ChemBridge database compound **ID: 5931690** and OSM database compound **ID: OSM-S-31** with predicted hERG inhibition potency of 1.86 nM and 4.7 μM, respectively (Figures 10, 11, respectively).

To access the IC_50_ for hERG inhibition we used a step ramp protocol (Crumb et al., 2016) as described in the Methods. Cisapride, 20 nM, was used as a positive control and all experiments were performed at ambient temperature. Drug solutions were prepared according to their predicted IC_50_ values from virtual screening protocol. For example, the predicted IC_50_ value of ChemBridge database (**Db ID: 5931690**) compound was 1.86 nM (Figure 10) therefore 1, 3, 100, 300, and 1,000 nM concentrations were used. The data in Figure 10C shows the selected sweeps at equilibrium after the application of different drug concentrations of compound ID: 5931690 and the corresponding dose-response curve is shown in Figure 10D. Similarly, for the OSM database compound (Williamson et al., 2016; **Db ID: OSM-S-31**), predicted IC_50_ value was 4.79 μM. Therefore, 1, 5, 10, and 30 μM different drug concentrations were used. Figure 11C shows the selected sweeps at equilibrium in response to each dose concentration of OSM-S-31.

The IC_50_ values of both compounds were calculated by fitting the Hill Equation (see Methods) to the dose-response curves for each drug. **Db ID: 5931690** and **Db ID: OSM-S-31** compounds showed experimental IC_50_ values of 71.4 nM and 3.89 μM as compared to predicted IC_50_ values of 1.86 nM and 4.79 μM, respectively. This reflects a difference of 1.6 log units for predicted hERG inhibitory values for **Db ID: 5931690**, and <0.1 log units difference for the predicted and measured IC_50_ values for **Db ID: OSM-S-31**.

## Discussion

In present investigation, combined ligands and structure based pharmacoinformatics protocol supported by patch-clamp electrophysiological experiments on selected hits, has been proposed to advocate hERG liability of new chemical entities (NCEs) during early stages of drug design and development. Recently, Siramsthetty et al. elucidated the impact of data quality, structural diversity and activity threshold of the training set and on the performance of various machine learning algorithms for the prediction of hERG liability (Siramshetty et al., 2018). This and many other previous reports reflect that inconsistent biological data under different experimental condition might result in noisy or unreliable predictive models (Zvinavashe et al., 2008; Su et al., 2010). Here, combined ligand- (GRIND) and structure based- (ligand protein interaction guided pharmacophore) pharmacoinformatics models developed using training and tests evaluated with uniform experimental protocol such as, experimental methods and cell lines may assist in overall robustness of the present hERG liability prediction approach.

Finally selected GRIND model illustrates a virtual receptor site of two hydrophobic, one hydrogen bond donor and two steric hotspot regions for the binding of present data sets of hERG blockers. One of the hydrophobic regions (DRY1: yellow hotspots, enclosed by a circle in Figure 7) identified by the GRIND model may represent the most crucial virtual receptor site as the distance of other contours including second hydrophobic region (DRY2), the steric molecular hotspot (TIP1) and a hydrogen bond donor (N1) contour has been calculated from this region. Thus, it is tempting to speculate that this hydrophobic region (DRY1) may provide an anchoring point for the snug fit of the ligand followed by conformational change of the other part of the ligand to find complementary interaction points within binding site of hERG. Additionally, our model also elucidates the importance of molecular shape of the hERG blockers by spotting two steric hotspot regions (TIP1 and TIP2) at the virtual receptor site of structurally diverse data set of hERG inhibitors. The hydrophobic contours of virtual receptor site in our GRIND model also complement the four concentric rings of Tyr_652 and Phe_656 amino acid residues in the central cavity of hERG. This is also supported by various reports about the role of Tyr_652 and Phe_656 residues in drug trapping (Fernandez et al., 2004; Kalyaanamoorthy and Barakat, 2017; Vandenberg et al., 2017).

Various studies also linked the tendency of a compound to get trapped inside the hERG channel with simple increment in the molecular weight or logP (Gleeson, 2008). However, in lead optimization programmes, there is a trend of increase in molecular weight and lipophilicity with an increase in biological activity against a therapeutic target which may offer greater hERG liability *in vitro*. Additionally, compounds that are trapped inside the hERG are transported through membrane bilayer via cytoplasm inside the hERG (Jabeen et al., 2012). A compound that may efficiently cross the biomembrane barrier may show more hERG inhibition as compared to those with suboptimal transport properties. Therefore, lipophilic efficiency (LipE) calculation in the present study may provide a straightforward way to normalize this effect and aids in identifying the hERG inhibitors (templates) with increased activity as a result of direct interaction with hERG rather than higher biomembrane distribution. Thus, in present study, ligand protein interaction guided hERG inhibition hypothesis by using templates with best IC_50_/lipophilicity and molecular IC_50_/molecular weight ratio may offer efficient models for the virtual screening of the new chemical entities.

Overall, our hERG inhibition pharmacophore hypothesis includes two aromatic, one hydrophobic and one hydrogen bond acceptor features which select highly potent hERG inhibitors (IC_50_ < 100 μM). Interestingly, a pairwise comparison of the 3D structural features of the final pharmacophore model with the GRIND contours depicting virtual receptor site revealed a great degree of complementarity as summarized in Table 4. For instance, DRY-DRY contours at a mutual distance of ~14 **A**° in virtual receptor space may complement two aromatic (Aro1 and Aro2) pharmacophore features at a distance of 6.1 **A**° within the template structure. This is further strengthen by a recent three point 3D-SDAR model (Stoyanova-Slavova et al., 2017) where two aromatic rings at a distance of a 4.5–11.5 **A**° have been identified important hERG toxicophore features. Similarly, DRY-N1 pair of hotspots depict a hydrophobic and a hydrogen bond donor region at a mutual distance of ~10 **A**° at the virtual receptor site may complement the hydrophobic and hydrogen bond acceptor group (HBA) within the template structure which further strengthens our proposed binding hypothesis of hERG inhibitors.

**Table 4:** Comparison between distances of pharmacophoric features of GRIND at VRS and pharmacophore model within the template.

**GRIND features and distances at VRS** ~**A°**		**Pharmacophore features and distances within template A°**	
DRY-DRY	14	Aromatic1-Aromatic 2 Hydrophobic-Aromatic1	6.1 5.1
DRY-N1	10	Hydrogen bond acceptor-Hydrophobic	5.6

Previously, many attempts have also been made to develop hERG inhibition hypothesis models based on 3D structural features of the variable training data (Cavalli et al., 2002; Ekins et al., 2002; Pearlstein et al., 2003; Aronov and Goldman, 2004; Testai et al., 2004; Aronov, 2006; Crumb et al., 2006; Johnson et al., 2007; Coi et al., 2008; Garg et al., 2008; Kramer et al., 2008; Shamovsky et al., 2008; Durdagi et al., 2011; Tan et al., 2012; Kratz et al., 2014; Wang et al., 2016; Chemi et al., 2017; Stoyanova-Slavova et al., 2017; Siramshetty et al., 2018; Wacker and Noskov, 2018). Already reported pharmacophore models delineated the presence of two or three hydrophobic or aromatic moieties (Cavalli et al., 2002; Ekins et al., 2002; Pearlstein et al., 2003; Aronov and Goldman, 2004; Aronov, 2006; Crumb et al., 2006; Coi et al., 2008; Garg et al., 2008; Kramer et al., 2008; Durdagi et al., 2011; Kratz et al., 2014). Additionally, various models identified basic nitrogen as a crucial pharmacophoric feature (Cavalli et al., 2002; Ekins et al., 2002; Pearlstein et al., 2003; Johnson et al., 2007; Coi et al., 2008; Kramer et al., 2008; Kratz et al., 2014) for hERG inhibition. Some authors also demonstrated the positive contribution of the hydrogen bond acceptor feature toward hERG inhibition potential in the closed state of the channel (Aronov and Goldman, 2004; Du-Cuny et al., 2011; Durdagi et al., 2011; Kratz et al., 2014). Furthermore, the impact of hydrogen bonding of various chemical scaffolds with amino acid residues Thr_623, Ser_624 and Val_625 within the central cavity has also been associated with hERG inhibition potential (Mitcheson and Perry, 2003; Aronov, 2005). Some other studies added that central cavity of the hERG may favors the binding of cationic drugs by amplifying the negative electrostatic potential within the central cavity that in turn may increase drug affinity (Aronov, 2005; Wang and MacKinnon, 2017).

Most of these models were able to predict hERG inhibition properties of a drug by assigning nominal class label (Inhibitor/Non-inhibitor: Yes/No). Some 2D QSAR models (Seierstad and Agrafiotis, 2006; Yoshida and Niwa, 2006) also predict numerical hERG inhibition values. However, the available 3D QSAR represent class specific features developed using small dataset of compounds due to alignment constraints of 3D QSAR methodologies (Ekins et al., 2002; Keserü, 2003; Pearlstein et al., 2003).

Although more recently, various machine learning models for the classification of large data sets based on 2D descriptors and fingerprints may offer good predictive ability and an automated potential to integrate with other conventional and modern modeling approaches (Siramshetty et al., 2018; Wacker and Noskov, 2018). Yet, a ligand and structure based integrated approach based on 3D descriptors information may also represent a promising route for toxicological profiling of new chemical entities. Therefore, we integrated novel alignment independent GRIND descriptors and conventional pharmacophore feature descriptors and propose a pharmacoinformatics strategy for the stepwise nominal (yes/no) as well as numeric (absolute) prediction of hERG inhibition potential (pIC_50_) of NCEs. Moreover, in the present investigation, an exhaustive data curation protocol has been adopted to select experimentally homogeneous training as well as test data. Moreover, compounds of the training set belong to 11 diverse therapeutic classes (see Supporting Information smiles.csv) hence cover a large and diverse chemical and structural space. However, GRiD Independent Descriptors represent a novel class of descriptors which are insensitive to structural alignment of the data and thus, have ability to predict chemically diverse data set of compounds as compared to conventional 3D QSAR descriptors (Pastor et al., 2000). Therefore, the applicability domain of the training data identified by principal component scores of GRIND variables cover a wide range from −5.8 to 6.2. Projection of test set I and test set II also occupy the same applicability domain (Figure 2). Also the principal component scores of GRIND variables of the 100 top predicted hits ranges from −5.0 to 5.0 as shown in Figure S7. Thus, it reflects the predictive ability of structurally diverse data set of hERG blockers. Methodologically, in the present study highly significant GRIND variables have been selected with the help of AMANDA algorithm (Durán et al., 2008) as described by Pastor et al. (Pastor et al., 2000) in comparison with ALMOND algorithm utilized by most of the previous investigations that select a fixed number of variable irrespective of strength of the MIFs. Furthermore, in present investigation more robust pharmacophore models have been developed by normalizing suboptimal transport factor of the data and selecting templates with best pIC_50_/clogP and pIC_50_/molecular weight ratio. Overall, our final models may offer hERG inhibitory potency predictions from higher nanomolar to lower micromolar range that is validated with the help of whole-cell patch clamp experiment.

Overall, partial least square analysis of final GRIND variables reflects a cross validated *q*^2^ of 0.63 and *r*^2^ of 0.68. Additionally, our final pharmacophore model showed 0.72 sensitivity and 0.75 specificity with 0.72 MCC. These reflect statistically significant model. Subsequently, experimental validation of these models using whole cell patch clamp technique delineates difference of 1.6 and <0.1 log units between experimentally determined and predicted hERG inhibitory (pIC_50_) values of selected hits **Db ID: 5931690**, and **Db ID: OSM-S-31** demonstrate excellent agreement in the prediction of μM range but underestimate the hERG inhibition potential in nM range. Thus, it seems that the numerical values of the hERG inhibition potential of the nanomolar active hit is not predicted accurately, but the model may still allow correct identification of activity trends (from nanomolar to micromolar). It is still of great value to know which molecules are the “best” (low activity against a hERG) and which are likely to have high hERG inhibition potential. This allows molecules to be prioritized for synthesis against respective therapeutic targets.

Although, the recent cryo-EM structure of the hERG in its open state of the channel may assist in probing structure guided hERG inhibition profiles of new and safer chemical entities (Wang and MacKinnon, 2017). However, the volume of the hydrophobic pockets in the recent hERG structure and their impact on the binding of drugs that are trapped in the closed state of hERG remain obscure. Though majority of highly potent hERG blockers including dofetilide, astemizole, terfenadine, and cisapride, show from 2 to 70-fold higher affinity for the inactivated state than to the open conformational state of the channel (Perrin et al., 2008). Thus, it stresses the need to determine electron microscopic/crystal structures of hERG in closed as well as in the open state of the channel bound with one of the prototype ligands such as, **Dofetilide, MK499**, or **E4031** to understand the molecular basis of drug binding within hERG. However, it is challenging mainly because membrane spanning hERG channel exhibit 4-fold symmetry that might result in negligible electron density due to asymmetric binding of a drug with its one out of four subunits. Therefore, in the absences of explicit experimental structural data, present investigation offers experimentally validated template-based pharmacophore models supported by statistically significant GRIND model to predict the hERG inhibition potential of diverse chemical scaffolds.

## Conclusion

Herein, we present a novel pharmacoinformatics strategy for the nominal as well as numerical prediction of hERG inhibition potential (pIC_50_) of NCEs during initial stages of drug development. GRid-Independent molecular Descriptor (GRIND) models have been developed to probe virtual receptor site representing most favorable interacting hotspots within the binding cavity of hERG. Furthermore, pharmacophore templates with best hERG pIC_50_/clogP and pIC_50_/molecular weight have been selected to normalize pIC_50_ values against hERG due to membrane distribution effect rather than direct interaction with hERG. Ligand-protein interaction profiles guided pharmacophore features of 13 selected templates in open and closed conformational state of hERG have been evaluated to probe 3D structural features of diverse data set hERG inhibitors. Overall, our final pharmacophore model revealed the presence of two aromatic one hydrophobic and one hydrogen bond acceptor group at particular mutual distance in most potent hERG inhibitors which complement the respective hydrophobic and hydrogen bond donor contours at the virtual receptor site produced by the final GRIND model. The maximum difference of ±1.6 log unit between actual and predicted hERG inhibition potential values of the selected hits **Db ID: 5931690** and **Db ID: OSM-S-31** further reflect the robustness of our virtual screening protocol and thus, could aid to prioritize NCEs according to hERG inhibition potential from nanomolar to micromolar range.

## Author contributions

IJ designed the research project for the Ph.D. degree of SM. SM and IJ carried out the build *in silico* models and analyzed results. JV and IJ designed the wet lab experiments. SM performed the experiments MW, JV, and AH analyzed the experimental data. SM and IJ wrote the manuscript. MT provided compounds for the validation. ET helped in resolving solubility issue of the OSM compounds. JV and IJ reviewed the paper.

### Conflict of interest statement

The authors declare that the research was conducted in the absence of any commercial or financial relationships that could be construed as a potential conflict of interest.

## Supplementary material

Data curration protocol, Pie chat, to show dataset distribution. Correlation plot, between IC50 values of compounds obtained from HEK and CHO cell line. PCA cluster I, II and III representative compounds. Conformational analysis for GRIND. Plots of, Lipophilic and Ligand efficiency profiling. Complete docking protocol and ligand-protein interaction diagrams of all selected compounds. Hit selection protocol. NMR spectra of compound OSM-S-31, and 5931690. Molecular Formula Strings (CSV) along with IC50 values are available as SMILES.csv.

## Abbreviations

hERG: human ether-a-go-go-related gene
TdPs: torsades de pointes
IC_50_: half maximal inhibitory concentration
NCE: new chemical entities
GRIND: GRid INdependent molecular Descriptor
LipE: lipophilic efficiency
LE: ligand efficiency
μM: micromolar
nM: nanomolar
*I*_*Kr*_: rectifier potassium current
ECG: electrocardiogram
ICH: international conference on harmonization
2D QSAR: 2-dimensional quantitative structure activity relationship
3D QSAR: 3-dimensional quantitative structure activity relationship
CoMSIA: comparative molecular similarity indices analysis
CoMFA: comparative molecular field analysis
hQSAR: hologram quantitative structure activity relationship
PCA: principal component analysis
MIFs: molecular interaction fields
VRS: virtual receptor site
PLS: partial least square analysis
LOO: leave one out
SDEP: standard deviation of error prediction
FFD: fractional factorial design
LV: latent variables
OSM: open source malaria
pIC_50_: log of inhibitory potency values
HEK: human embryonic kidney
CHO: chinese hamster ovary
CLACC: consistently large auto and cross-correlation
FQ: fit quality

## References

[B1] Alvarez P. A. Pahissa J. (2010). QT alterations in psychopharmacology: proven candidates and suspects. Curr. Drug Saf. 5, 97–104. 10.2174/157488610789869265 20210726

[B2] Alves V. M. Golbraikh A. Capuzzi S. J. Liu K. Lam W. I. Korn D. (2018). Multi-descriptor read across (MuDRA): a simple and transparent approach for developing accurate QSAR models. J Chem Inf Model. 58, 1214–1223. 10.1021/acs.jcim.8b00124 29809005 PMC7917006

[B3] Aronov A. M. (2005). Predictive *in silico* modeling for hERG channel blockers. Drug Discov. Today 10, 149–155. 10.1016/S1359-6446(04)03278-7 15718164

[B4] Aronov A. M. (2006). Common pharmacophores for uncharged human ether-a-go-go-related gene (hERG) blockers. J. Med. Chem. 49, 6917–6921. 10.1021/jm060500o 17154521

[B5] Aronov A. M. Goldman B. B. (2004). A model for identifying HERG K+ channel blockers. Bioorg. Med. Chem. 12, 2307–2315. 10.1016/j.bmc.2004.02.003 15080928

[B6] Bains W. Basman A. White C. (2004). HERG binding specificity and binding site structure: evidence from a fragment-based evolutionary computing SAR study. Prog. Biophys. Mol. Biol. 86, 205–233. 10.1016/j.pbiomolbio.2003.09.001 15288759

[B7] Baroni M. Costantino G. Cruciani G. Riganelli D. Valigi R. Clementi S. (1993). Generating optimal linear PLS estimations (GOLPE): an advanced chemometric tool for handling 3D-QSAR problems. Quant. Struct. Act. Relat. 12, 9–20. 10.1002/qsar.19930120103

[B8] Barry P. H. (1994). JPCalc, a software package for calculating liquid junction potential corrections in patch-clamp, intracellular, epithelial and bilayer measurements and for correcting junction potential measurements. J. Neurosci. Methods 51, 107–116. 10.1016/0165-0270(94)90031-0 8189746

[B9] Bento A. P. Gaulton A. Hersey A. Bellis L. J. Chambers J. Davies M.. (2013). The ChEMBL bioactivity database: an update. Nucleic Acids Res. 42, D1083–D1090. 10.1093/nar/gkt1031 24214965 PMC3965067

[B10] Bischoff U. Schmidt C. Netzer R. Pongs O. (2000). Effects of fluoroquinolones on HERG currents. Eur. J. Pharmacol. 406, 341–343. 10.1016/S0014-2999(00)00693-2 11040340

[B11] Caron G. Ermondi G. (2007). Influence of conformation on GRIND-based three-dimensional quantitative structure–activity relationship (3D-QSAR). J. Med. Chem. 50, 5039–5042. 10.1021/jm0704651 17760433

[B12] Cavalli A. Poluzzi E. De Ponti F. Recanatini M. (2002). Toward a pharmacophore for drugs inducing the long QT syndrome: insights from a CoMFA study of HERG K+ channel blockers. J. Med. Chem. 45, 3844–3853. 10.1021/jm0208875 12190308

[B13] Chaudhary K. W. O'Neal J. M. Mo Z.-L. Fermini B. Gallavan R. H. Bahinski A. (2006). Evaluation of the rubidium efflux assay for preclinical identification of HERG blockade. Assay Drug Dev. Technol. 4, 73–82. 10.1089/adt.2006.4.73 16506891

[B14] Chavan S. Abdelaziz A. Wiklander J. G. Nicholls I. A. (2016). A k-nearest neighbor classification of hERG K+ channel blockers. J. Comput. Aided Mol. Des. 30, 229–236. 10.1007/s10822-016-9898-z 26860111 PMC4802000

[B15] Chemi G. Gemma S. Campiani G. Brogi S. Butini S. Brindisi M. (2017). Computational tool for fast *in silico* evaluation of hERG K+ channel affinity. Front. Chem. 5:7. 10.3389/fchem.2017.00007 28503546 PMC5408157

[B16] Chiu P. J. Marcoe K. F. Bounds S. E. Lin C.-H. Feng J.-J. Lin A. (2004). Validation of a [3H] astemizole binding assay in HEK293 cells expressing HERG K+ channels. J. Pharmacol. Sci. 95, 311–319. 10.1254/jphs.FPE0040101 15272206

[B17] Choe H. Nah K. H. Lee S. N. Lee H. S. Lee H. S. Jo S. H.. (2006). A novel hypothesis for the binding mode of HERG channel blockers. Biochem. Biophys. Res. Commun. 344, 72–78. 10.1016/j.bbrc.2006.03.146 16616004

[B18] Coi A. Massarelli I. Testai L. Calderone V. Bianucci A. M. (2008). Identification of toxicophoric features for predicting drug-induced QT interval prolongation. Eur. J. Med. Chem. 43, 2479–2488. 10.1016/j.ejmech.2007.12.025 18262683

[B19] Crumb W. J. Ekins S. Sarazan R. D. Wikel J. H. Wrighton S. A. Carlson C. (2006). Effects of antipsychotic drugs on Ito, INa, Isus, IK1, and hERG: QT prolongation, structure activity relationship, and network analysis. Pharm. Res. 23, 1133–1143. 10.1007/s11095-006-0070-7 16715368

[B20] Crumb W. J. Vicente J. Johannesen L. Strauss D. G. (2016). An evaluation of 30 clinical drugs against the comprehensive *in vitro* proarrhythmia assay (CiPA) proposed ion channel panel. J. Pharm. Toxicol. Methods 81, 251–262. 10.1016/j.vascn.2016.03.009 27060526

[B21] De Bruin M. Pettersson M. Meyboom R. Hoes A. Leufkens H. (2005). Anti-HERG activity and the risk of drug-induced arrhythmias and sudden death. Eur. Heart J. 590–597. 10.1093/eurheartj/ehi092 15637086

[B22] De Ponti F. Poluzzi E. Cavalli A. Recanatini M. Montanaro N. (2002). Safety of non-antiarrhythmic drugs that prolong the QT interval or induce torsade de pointes. Drug Saf. 25, 263–286. 10.2165/00002018-200225040-00004 11994029

[B23] Dempsey C. E. Wright D. Colenso C. K. Sessions R. B. Hancox J. C. (2014). Assessing hERG pore models as templates for drug docking using published experimental constraints: the inactivated state in the context of drug block. J. Chem. Inf. Model. 54, 601–612. 10.1021/ci400707h 24471705 PMC3977586

[B24] Dubus E. Ijjaali I. Petitet F. Michel A. (2006). *In silico* classification of hERG channel blockers: a knowledge-based strategy. ChemMedChem 1, 622–630. 10.1002/cmdc.200500099 16892402

[B25] Du-Cuny L. Chen L. Zhang S. (2011). A critical assessment of combined ligand-and structure-based approaches to HERG channel blocker modeling. J. Chem. Inf. Model. 51, 2948–2960. 10.1021/ci200271d 21902220 PMC3894065

[B26] Durán Alcaide Á. (2010). Development of High-Performance Algorithms for a New Generation of Versatile Molecular Descriptors. Barcelona: The Pentacle software, Universitat Pompeu abra.

[B27] Durán A. Martínez G. C. Pastor M. (2008). Development and validation of AMANDA, a new algorithm for selecting highly relevant regions in molecular interaction fields. J. Chem. Inf. Model. 48, 1813–1823. 10.1021/ci800037t 18693718

[B28] Durdagi S. Duff H. J. Noskov S. Y. (2011). Combined receptor and ligand-based approach to the universal pharmacophore model development for studies of drug blockade to the hERG1 pore domain. J. Chem. Inf. Model. 51, 463–474. 10.1021/ci100409y 21241063

[B29] Ekins S. Crumb W. J. Sarazan R. D. Wikel J. H. Wrighton S. A. (2002). Three-dimensional quantitative structure-activity relationship for inhibition of human ether-a-go-go-related gene potassium channel. J. Pharm. Exp. Ther. 301, 427–434. 10.1124/jpet.301.2.427 11961040

[B30] Elisseeff A. Pontil M. (2003). Leave-one-out error and stability of learning algorithms with applications. NATO Science Series Sub Series III Comput. Syst. Sci. 190, 111–130.

[B31] Farid R. Day T. Friesner R. A. Pearlstein R. A. (2006). New insights about HERG blockade obtained from protein modeling, potential energy mapping, and docking studies. Bioorg. Med. Chem. 14, 3160–3173. 10.1016/j.bmc.2005.12.032 16413785

[B32] Ferguson D. M. Raber D. J. (1989). A new approach to probing conformational space with molecular mechanics: random incremental pulse search. J. Am. Chem. Soc. 111, 4371–4378. 10.1021/ja00194a034

[B33] Fernandez D. Ghanta A. Kauffman G. W. Sanguinetti M. C. (2004). Physicochemical features of the HERG channel drug binding site. J. Biol. Chem. 279, 10120–10127. 10.1074/jbc.M310683200 14699101

[B34] Finlayson K. Turnbull L. January C. T. Sharkey J. Kelly J. S. (2001). [3 H] Dofetilide binding to HERG transfected membranes: a potential high throughput preclinical screen. Eur. J. Pharmacol. 430, 147–148. 10.1016/S0014-2999(01)01362-0 11698075

[B35] Food and Drug Administration H. (2001). International Conference on Harmonisation; guidance on S7A safety pharmacology studies for human pharmaceuticals; availability. Notice. Fed. Regist. 66, 36791. 12356097

[B36] Food and Drug Administration H. (2005a). International Conference on Harmonisation; guidance on E14 clinical evaluation of QT/QTc interval prolongation and proarrhythmic potential for non-antiarrhythmic drugs; availability. Notice. Fed. Regist. 70, 61134. 16237860

[B37] Food and Drug Administration H. (2005b). International Conference on Harmonisation; guidance on S7B nonclinical evaluation of the potential for delayed ventricular repolarization (QT interval prolongation) by human pharmaceuticals; availability. Notice. Fed. Regist. 70, 61133. 16237859

[B38] Freeman-Cook K. D. Hoffman R. L. Johnson T. W. (2013). Lipophilic efficiency: the most important efficiency metric in medicinal chemistry. Fut. Med. Chem. 5, 113–115. 10.4155/fmc.12.208 23360135

[B39] Garg D. Gandhi T. Mohan C. G. (2008). Exploring QSTR and toxicophore of hERG K+ channel blockers using GFA and HypoGen techniques. J. Mol. Graph. Model. 26, 966–976. 10.1016/j.jmgm.2007.08.002 17928249

[B40] Gasteiger J. Rudolph C. Sadowski J. (1990). Automatic generation of 3D-atomic coordinates for organic molecules. Tetrahedron Comput. Methodol. 3, 537–547. 10.1016/0898-5529(90)90156-3

[B41] Gill P. E. Murray W. Wright M. H. (1981). Practical Optimization. Bradford, UK.

[B42] Gleeson M. P. (2008). Generation of a set of simple, interpretable ADMET rules of thumb. J. Med. Chem. 51, 817–834. 10.1021/jm701122q 18232648

[B43] Groom C. R. Bruno I. J. Lightfoot M. P. Ward S. C. (2016). The Cambridge structural database. Acta Crystallogr. Sect. B Struct. Sci. Cryst. Eng. Mater. 72, 171–179. 10.1107/S2052520616003954 27048719 PMC4822653

[B44] Guth B. D. (2007). Preclinical cardiovascular risk assessment in modern drug development. Toxicol. Sci. 97, 4–20. 10.1093/toxsci/kfm026 17351262

[B45] Halgren T. A. (1996). Merck molecular force field. I. Basis, form, scope, parameterization, and performance of MMFF94. J. Comput. Chem. 17, 490–519. 10.1002/(SICI)1096-987X(199604)17:5/6<490::AID-JCC1>3.0.CO;2-P

[B46] Hamill O. P. Marty A. Neher E. Sakmann B. Sigworth F. (1981). Improved patch-clamp techniques for high-resolution current recording from cells and cell-free membrane patches. Pflügers Archiv Eur. J. Physiol. 391, 85–100. 10.1007/BF00656997 6270629

[B47] Hancox J. C. McPate M. J. El Harchi A. Hong Zhang Y. (2008). The hERG potassium channel and hERG screening for drug-induced torsades de pointes. Pharmacol. Ther. 119, 118–132. 10.1016/j.pharmthera.2008.05.009 18616963

[B48] Hill A. P. Perrin M. J. Heide J. Campbell T. J. Mann S. A. Vandenberg J. I. (2014). Kinetics of drug interaction with the Kv11. 1 potassium channel. Mol. Pharmacol. 85, 769–776. 10.1124/mol.114.091835 24586056

[B49] Hopkins A. L. Groom C. R. Alex A. (2004). Ligand efficiency: a useful metric for lead selection. Drug Discov. Today 9, 430–431. 10.1016/S.1359-6446(04)03069-7 15109945

[B50] I C. C. G. (2013). Molecular Operating Environment (MOE). Montreal, QC: Chemical Computing Group Inc.

[B51] Jabeen I. Pleban K. Rinner U. Chiba P. Ecker G. F. (2012). Structure–activity relationships, ligand efficiency, and lipophilic efficiency profiles of benzophenone-type inhibitors of the multidrug transporter P-glycoprotein. J. Med. Chem. 55, 3261–3273. 10.1021/jm201705f 22452412 PMC3326594

[B52] Johnson S. R. Yue H. Conder M. L. Shi H. Doweyko A. M. Lloyd J.. (2007). Estimation of hERG inhibition of drug candidates using multivariate property and pharmacophore SAR. Bioorg. Med. Chem. 15, 6182–6192. 10.1016/j.bmc.2007.06.028 17596950

[B53] Jurkiewicz N. K. Sanguinetti M. C. (1993). Rate-dependent prolongation of cardiac action potentials by a methanesulfonanilide class III antiarrhythmic agent. Specific block of rapidly activating delayed rectifier K+ current by dofetilide. Circ. Res. 72, 75–83. 10.1161/01.RES.72.1.75 8417848

[B54] Kalyaanamoorthy S. Barakat K. H. (2017). Development of safe drugs: the hERG challenge. Med. Res. Rev. 38, 525–555. 10.1002/med.21445 28467598

[B55] Keserü G. M. (2003). Prediction of hERG potassium channel affinity by traditional and hologram qSAR methods. Bioorg. Med. Chem. Lett. 13, 2773–2775. 10.1016/S0960-894X(03)00492-X 12873512

[B56] Kiralj R. Ferreira M. (2009). Basic validation procedures for regression models in QSAR and QSPR studies: theory and application. J. Braz. Chem. Soc. 20, 770–787. 10.1590/S0103-50532009000400021

[B57] Kireeva N. Kuznetsov S. Bykov A. Tsivadze A. Yu (2013). Towards *in silico* identification of the human ether-a-go-go-related gene channel blockers: discriminative vs. generative classification models. SAR and QSAR. Environ. Res. 24, 103–117. 10.1080/1062936X.2012.742135 23152964

[B58] Kramer C. Beck B. Kriegl J. M. Clark T. (2008). A composite model for hERG blockade. ChemMedChem 3, 254–265. 10.1002/cmdc.200700221 18061919

[B59] Kratz J. M. Schuster D. Edtbauer M. Saxena P. Mair C. E. Kirchebner J.. (2014). Experimentally validated hERG pharmacophore models as cardiotoxicity prediction tools. J. Chem. Inf. Model. 54, 2887–2901. 10.1021/ci5001955 25148533

[B60] Lee W. Mann S. A. Windley M. J. Imtiaz M. S. Vandenberg J. I. Hill A. P. (2016). *In silico* assessment of kinetics and state dependent binding properties of drugs causing acquired LQTS. Prog. Biophys. Mol. Biol. 120, 89–99. 10.1016/j.pbiomolbio.2015.12.005 26713558

[B61] Lees-Miller J. P. Duan Y. Teng G. Q. Duff H. J. (2000). Molecular determinant of high-affinity dofetilide binding toHERG1 expressed in Xenopus oocytes: involvement of S6 sites. Mol. Pharmacol. 57, 367–374. 10648647

[B62] Leeson P. D. Springthorpe B. (2007). The influence of drug-like concepts on decision-making in medicinal chemistry. Nat. Rev. Drug Discov. 6, 881–890. 10.1038/nrd2445 17971784

[B63] Li Q. Jørgensen F. S. Oprea T. Brunak S. Taboureau O. (2008). hERG classification model based on a combination of support vector machine method and GRIND descriptors. Mol. Pharm. 5, 117–127. 10.1021/mp700124e 18197627

[B64] Li X. Zhang Y. Li H. Zhao Y. (2017). Modeling of the hERG K+ channel blockage using online chemical database and modeling environment (OCHEM). Mol. Inf. 36, 1700074. 10.1002/minf.201700074 28857516

[B65] Lipinski C. A. Lombardo F. Dominy B. W. Feeney P. J. (2012). Experimental and computational approaches to estimate solubility and permeability in drug discovery and development settings. Adv. Drug Deliv. Rev. 64, 4–17. 10.1016/j.addr.2012.09.019 11259830

[B66] Liu L.-L. Lu J. Lu Y. Zheng M.-Y. Luo X.-M. Zhu W.-L.. (2014). Novel Bayesian classification models for predicting compounds blocking hERG potassium channels. Acta Pharmacol. Sin. 35, 1093–1102. 10.1038/aps.2014.35 24976154 PMC4125710

[B67] Lu J. Lu D. Fu Z. Zheng M. Luo X. (2018). Machine learning-based modeling of drug toxicity. Comput. Syst. Biol. 247–264. 10.1007/978-1-4939-7717-8_15 29536448

[B68] Mannhold R. Kubinyi H. Folkers G. (2006). Molecular Interaction Fields: Applications in Drug Discovery and ADME Prediction. Darmstadt: John Wiley & Sons.

[B69] Mitcheson J. Perry M. (2003). Molecular determinants of high-affinity drug binding to HERG channels. Curr. Opin. Drug Discov. Dev. 6, 667–674. 14579516

[B70] Mitcheson J. S. Chen J. Lin M. Culberson C. Sanguinetti M. C. (2000). A structural basis for drug-induced long QT syndrome. Proc. Natl. Acad. Sci. U.S.A. 97, 12329–12333. 10.1073/pnas.210244497 11005845 PMC17341

[B71] Nosten F. Ter Kuile F. Luxemburger C. Woodrow C. Chongsuphajaisiddhi T. White N.. (1993). Cardiac effects of antimalarial treatment with halofantrine. Lancet 341, 1054–1056. 10.1016/0140-6736(93)92412-M 8096959

[B72] Pastor M. Cruciani G. McLay I. Pickett S. Clementi S. (2000). GRid-INdependent descriptors (GRIND): a novel class of alignment-independent three-dimensional molecular descriptors. J. Med. Chem. 43, 3233–3243. 10.1021/jm000941m 10966742

[B73] Pearlstein R. A. Vaz R. J. Kang J. Chen X.-L. Preobrazhenskaya M. Shchekotikhin A. E.. (2003). Characterization of HERG potassium channel inhibition using CoMSiA 3D QSAR and homology modeling approaches. Bioorg. Med. Chem. Lett. 13, 1829–1835. 10.1016/S0960-894X(03)00196-3 12729675

[B74] Perrin M. J. Kuchel P. W. Campbell T. J. Vandenberg J. I. (2008). Drug binding to the inactivated state is necessary but not sufficient for high-affinity binding to human ether-a-go-go-related gene channels. Mol. Pharmacol. 74, 1443–1452. 10.1124/mol.108.049056 18701618

[B75] Polak S. Wiśniowska B. Brandys J. (2009). Collation, assessment and analysis of literature *in vitro* data on hERG receptor blocking potency for subsequent modeling of drugs' cardiotoxic properties. J. Appl. Toxicol. 29, 183–206. 10.1002/jat.1395 18988205

[B76] Raschi E. Ceccarini L. De Ponti F. Recanatini M. (2009). hERG-related drug toxicity and models for predicting hERG liability and QT prolongation. Expert Opin. Drug Metab. Toxicol. 5, 1005–1021. 10.1517/17425250903055070 19572824

[B77] Raschi E. Vasina V. Poluzzi E. De Ponti F. (2008). The hERG K+ channel: target and antitarget strategies in drug development. Pharmacol. Res. 57, 181–195. 10.1016/j.phrs.2008.01.009 18329284

[B78] Redfern W. Carlsson L. Davis A. Lynch W. MacKenzie I. Palethorpe S.. (2003). Relationships between preclinical cardiac electrophysiology, clinical QT interval prolongation and torsade de pointes for a broad range of drugs: evidence for a provisional safety margin in drug development. Cardiovasc. Res. 58, 32–45. 10.1016/S0008-6363(02)00846-5 12667944

[B79] Reynolds C. H. Bembenek S. D. Tounge B. A. (2007). The role of molecular size in ligand efficiency. Bioorg. Med. Chem. Lett. 17, 4258–4261. 10.1016/j.bmcl.2007.05.038 17532632

[B80] Roden D. M. (2004). Drug-induced prolongation of the QT interval. N. Engl. J. Med. 350, 1013–1022. 10.1056/NEJMra032426 14999113

[B81] Roden D. M. Woosley R. L. Primm R. K. (1986). Incidence and clinical features of the quinidine-associated long QT syndrome: implications for patient care. Am. Heart J. 111, 1088–1093. 10.1016/0002-8703(86)90010-4 3716982

[B82] Sǎnchez-Chapula J. A. Ferrer T. Navarro-Polanco R. A. Sanguinetti M. C. (2003). Voltage-dependent profile of humanether-a-go-go-related gene channel block is influenced by a single residue in the S6 transmembrane domain. Mol. Pharmacol. 63, 1051–1058. 10.1124/mol.63.5.1051 12695533

[B83] Sanguinetti M. C. Jiang C. Curran M. E. Keating M. T. (1995). A mechanistic link between an inherited and an acquird cardiac arrthytmia: HERG encodes the IKr potassium channel. Cell 81, 299–307. 10.1016/0092-8674(95)90340-2 7736582

[B84] Sauer A. J. Newton-Cheh C. (2012). Clinical and genetic determinants of torsade de pointes risk. Circulation 125, 1684. 10.1161/CIRCULATIONAHA.111.080887 22474311 PMC3347483

[B85] Seierstad M. Agrafiotis D. K. (2006). A QSAR model of hERG binding using a large, diverse, and internally consistent training set. Chem. Biol. Drug Des. 67, 284–296. 10.1111/j.1747-0285.2006.00379.x 16629826

[B86] Shamovsky I. Connolly S. David L. Ivanova S. Nordén B. Springthorpe B.. (2008). Overcoming undesirable HERG potency of chemokine receptor antagonists using baseline lipophilicity relationships. J. Med. Chem. 51, 1162–1178. 10.1021/jm070543k 18257512

[B87] Sherman W. Day T. Jacobson M. P. Friesner R. A. Farid R. (2006). Novel procedure for modeling ligand/receptor induced fit effects. J. Med. Chem. 49, 534–553. 10.1021/jm050540c 16420040

[B88] Siramshetty V. B. Chen Q. Devarakonda P. Preissner R. (2018). The Catch-22 of predicting hERG blockade using publicly accessible bioactivity data. J. Chem. Inf. Model. 58, 1224–1233. 10.1021/acs.jcim.8b00150 29772901

[B89] Song M. Clark M. (2006). Development and evaluation of an *in silico* model for hERG binding. J. Chem. Inf. Model. 46, 392–400. 10.1021/ci050308f 16426073

[B90] Stansfeld P. J. Gedeck P. Gosling M. Cox B. Mitcheson J. S. Sutcliffe M. J. (2007). Drug block of the hERG potassium channel: insight from modeling. Proteins Struct. Funct. Bioinf. 68, 568–580. 10.1002/prot.21400 17444521

[B91] Stoyanova-Slavova I. B. Slavov S. H. Buzatu D. A. Beger R. D. Wilkes J. G. (2017). 3D-SDAR modeling of hERG potassium channel affinity: a case study in model design and toxicophore identification. J. Mol. Graph. Model. 72, 246–255. 10.1016/j.jmgm.2017.01.012 28129595

[B92] Su B.-H. Shen M.-Y. Esposito E. X. Hopfinger A. J. Tseng Y. J. (2010). *In silico* binary classification QSAR models based on 4D-fingerprints and MOE descriptors for prediction of hERG blockage. J. Chem. Inf. Model. 50, 1304–1318. 10.1021/ci100081j 20565102

[B93] Sun H. (2006). An accurate and interpretable Bayesian classification model for prediction of hERG liability. ChemMedChem 1, 315–322. 10.1002/cmdc.200500047 16892366

[B94] Sun H. Huang R. Xia M. Shahane S. Southall N. Wang Y. (2017). Prediction of hERG liability–using SVM classification, bootstrapping and jackknifing. Mol. Inf. 36, 1600126. 10.1002/minf.201600126 28000393 PMC5382096

[B95] Sun H. Xia M. Shahane S. A. Jadhav A. Austin C. P. Huang R. (2013). Are hERG channel blockers also phospholipidosis inducers? Bioorg. Med. Chem. Lett. 23, 4587–4590. 10.1016/j.bmcl.2013.06.034 23856051 PMC3736554

[B96] Tan Y. Chen Y. You Q. Sun H. Li M. (2012). Predicting the potency of hERG K+ channel inhibition by combining 3D-QSAR pharmacophore and 2D-QSAR models. J. Mol. Model. 18, 1023–1036. 10.1007/s00894-011-1136-y 21660488

[B97] Tang W. Kang J. Wu X. Rampe D. Wang L. Shen H.. (2001). Development and evaluation of high throughput functional assay methods for HERG potassium channel. J. Biomol. Screening 6, 325–331. 10.1177/108705710100600506 11689132

[B98] Testai L. Bianucci A. Massarelli I. Breschi M. Martinotti E. Calderone V. (2004). Torsadogenic cardiotoxicity of antipsychotic drugs: a structural feature, potentially involved in the interaction with cardiac HERG potassium channels. Curr. Med. Chem. 11, 2691–2706. 10.2174/0929867043364351 15544470

[B99] Thai K.-M. Ecker G. F. (2008a). A binary QSAR model for classification of hERG potassium channel blockers. Bioorg. Med. Chem. 16, 4107–4119. 10.1016/j.bmc.2008.01.017 18243713

[B100] Thai K. M. Ecker G. F. (2008b). Classification Models for hERG inhibitors by counter-propagation neural networks. Chem. Biol. Drug Des. 72, 279–289. 10.1111/j.1747-0285.2008.00705.x 18844674

[B101] Vandenberg J. I. Perozo E. Allen T. W. (2017). Towards a structural view of drug binding to hERG K+ channels. Trends Pharmacol. Sci. 38, 899–907. 10.1016/j.tips.2017.06.004 28711156 PMC6658208

[B102] Vandenberg J. I. Perry M. D. Perrin M. J. Mann S. A. Ke Y. Hill A. P. (2012). hERG K+ channels: structure, function, and clinical significance. Physiol. Rev. 92, 1393–1478. 10.1152/physrev.00036.2011 22988594

[B103] Wacker S. Noskov S. Y. (2018). Performance of machine learning algorithms for qualitative and quantitative prediction drug blockade of hERG1 channel. Comput. Toxicol. 6, 55–63. 10.1016/j.comtox.2017.05.001 29806042 PMC5967266

[B104] Walker B. Singleton C. Bursill J. Wyse K. Valenzuela S. Qiu M.. (1999). Inhibition of the human ether-a-go-go-related gene (HERG) potassium channel by cisapride: affinity for open and inactivated states. Br. J. Pharmacol. 128, 444–450. 10.1038/sj.bjp.0702774 10510456 PMC1571630

[B105] Wang S. Sun H. Liu H. Li D. Li Y. Hou T. (2016). ADMET evaluation in drug discovery. 16. Predicting hERG blockers by combining multiple pharmacophores and machine learning approaches. Mol. Pharm. 13, 2855–2866. 10.1021/acs.molpharmaceut.6b00471 27379394

[B106] Wang W. MacKinnon R. (2017). Cryo-EM structure of the open human ether-à-go-go-related K+ channel hERG. Cell 169, 422–430.e410. 10.1016/j.cell.2017.03.048 28431243 PMC5484391

[B107] Williamson A. E. Ylioja P. M. Robertson M. N. Antonova-Koch Y. Avery V. Baell J. B.. (2016). Open source drug discovery: highly potent antimalarial compounds derived from the tres cantos arylpyrroles. ACS Cent. Sci. 2, 687–701. 10.1021/acscentsci.6b00086 27800551 PMC5084075

[B108] Windley M. J. Lee W. Vandenberg J. I. Hill A. P. (2018). The temperature dependence of kinetics associated with drug block of hERG channels are compound specific and an important factor for proarrhythmic risk prediction. Mol. Pharmacol. 94, 760–769. 10.1124/mol.117.111534 29728448

[B109] Wold S. Esbensen K. Geladi P. (1987). Principal component analysis. Chemometr. Intell. Lab. Syst. 2, 37–52. 10.1016/0169-7439(87)80084-9

[B110] Yang H. T. Sun C. F. Cui C. C. Xue X. L. Zhang A. F. Li H. B. (2009). HERG-F463L potassium channels linked to long QT syndrome reduce IKr current by a trafficking-deficient mechanism. Clin. Exp. Pharmacol. Physiol. 36, 822–827. 10.1111/j.1440-1681.2009.05150.x 19215240

[B111] Yoshida K. Niwa T. (2006). Quantitative structure– activity relationship studies on inhibition of hERG potassium channels. J. Chem. Inf. Model. 46, 1371–1378. 10.1021/ci050450g 16711756

[B112] Zhang A. Sun C. Zhang L. Lv Y. Xue X. Li G.. (2013). L539 fs/47, a truncated mutation of human ether-a-go-go-related gene (hERG), decreases hERG ion channel currents in HEK 293 cells. Clin. Exp. Pharmacol. Physiol. 40, 28–36. 10.1111/1440-1681.12028 23134353

[B113] Zhou Z. Vorperian V. R. Gong Q. Zhang S. January C. T. (1999). Block of HERG potassium channels by the antihistamine astemizole and its metabolites desmethylastemizole and norastemizole. J. Cardiovasc. Electrophysiol. 10, 836–843. 10.1111/j.1540-8167.1999.tb00264.x 10376921

[B114] Zvinavashe E. Murk A. J. Rietjens I. M. (2008). Promises and pitfalls of quantitative structure–activity relationship approaches for predicting metabolism and toxicity. Chem. Res. Toxicol. 21, 2229–2236. 10.1021/tx800252e 19548346

