# Supplementary material for: Experimentally Validated Pharmacoinformatics Approach to Predict hERG Inhibition Potential of New Chemical Entities
Source: Front Pharmacol. 2018 Sep 19;9:1035. doi: 10.3389/fphar.2018.01035 (PMC6176658; doi:10.3389/fphar.2018.01035)
Supplement: Supplementary file 4 [file Data_Sheet_2.docx]

**Supporting Information**

Experimentally Validated Pharmacoinformatics Approach to Predict hERG Inhibition Potential of New Chemical Entities

**Authors:** Saba Munawar^1, 2,^ Monique J. Windley^2^, Adam P. Hill^2^, Edwin G. Tse^3^, Matthew H. Todd^3^, Jamie I. Vandenberg^2^, Ishrat Jabeen^1^*

**Affiliations:**

1. Research Center for Modeling and Simulation (RCMS), National University of Science and Technology, Sector H-12, 44000, Islamabad Pakistan.
2. Victor Chang Cardiac Research Institute, Lowy Packer Building, 405 Liverpool Street Darlinghurst Sydney NSW 2010, Australia
3. School of Chemistry, The University of Sydney, F11, Eastern Ave, Sydney NSW 2006, Australia

**Corresponding Author:**

**Dr. Ishrat Jabeen** Associate Professor. *E-mail: [ishrat.jabeen@rcms.nust.edu.pk](mailto:ishrat.jabeen@rcms.nust.edu.pk) Phone: +92-51-90855732. Research Center for Modeling and Simulation (RCMS). National University of Sciences and Technology (NUST). Sector H-12, Islamabad, Pakistan

**Contents**

**Material and Method**

| **Figure S1** | **Pie chart representing dataset distribution of hERG inhibitors from different sources** |
| --- | --- |
| **Figure S2** | **Data curation protocol** |
| **Figure S3** | **Correlation plot between HEK and CHO cell line studied on whole cell patch clamp technique** |
| **Table 1** | **Comparison of pIC_50_ values of compounds taken from literature of HEK and CHO cell line by applying whole-cell patch clamp technique** |
| **Figure S4** | **Template selection protocol: Lipophilic and Ligand efficiency profile of hERG inhibitors** |
| **Figure S5** | **Molecular docking and pose selection protocol** |
| **Figure S6**  **Figure S7** | **Hit selection protocol for experimental validation**  PCA analysis of hit selection for experimental validation |
| **Figure S8** | **NMR spectra of ChemBridge compound Db ID: 5931690** |
| **Figure S9** | **NMR spectra of OSM compound Db ID: OSM-S-31** |

**Result**

| **Figure S10** | **PCA Correlogram and representative compound of each cluster** |
| --- | --- |
| **Figure S11** | **MK499 ligand protein interaction in open and closed conformational state of hERG** |
| **Figure S12** | **Dofetilide, 9-hydroxy Risperidone and Haloperidol ligand protein interaction in open and closed conformational state of hERG** |
| **Figure S13** | **E4031, Trimethoprim, Droperidol ligand protein interaction in open and closed conformational state of hERG** |
| **Figure S14** | **Benperidol, Norastemizol, vesnarinone ligand protein interaction in open and closed conformational state of hERG** |
| **Figure S15** | **BMLC_1835_4, Risperidone, glycerol nonivamide ligand protein interaction in open and closed conformational state of hERG** |

**Material and Method**

**Dataset Collection and Refinement**

**Figure S1.** Pie chart representing dataset distribution of hERG blockers from different sources.


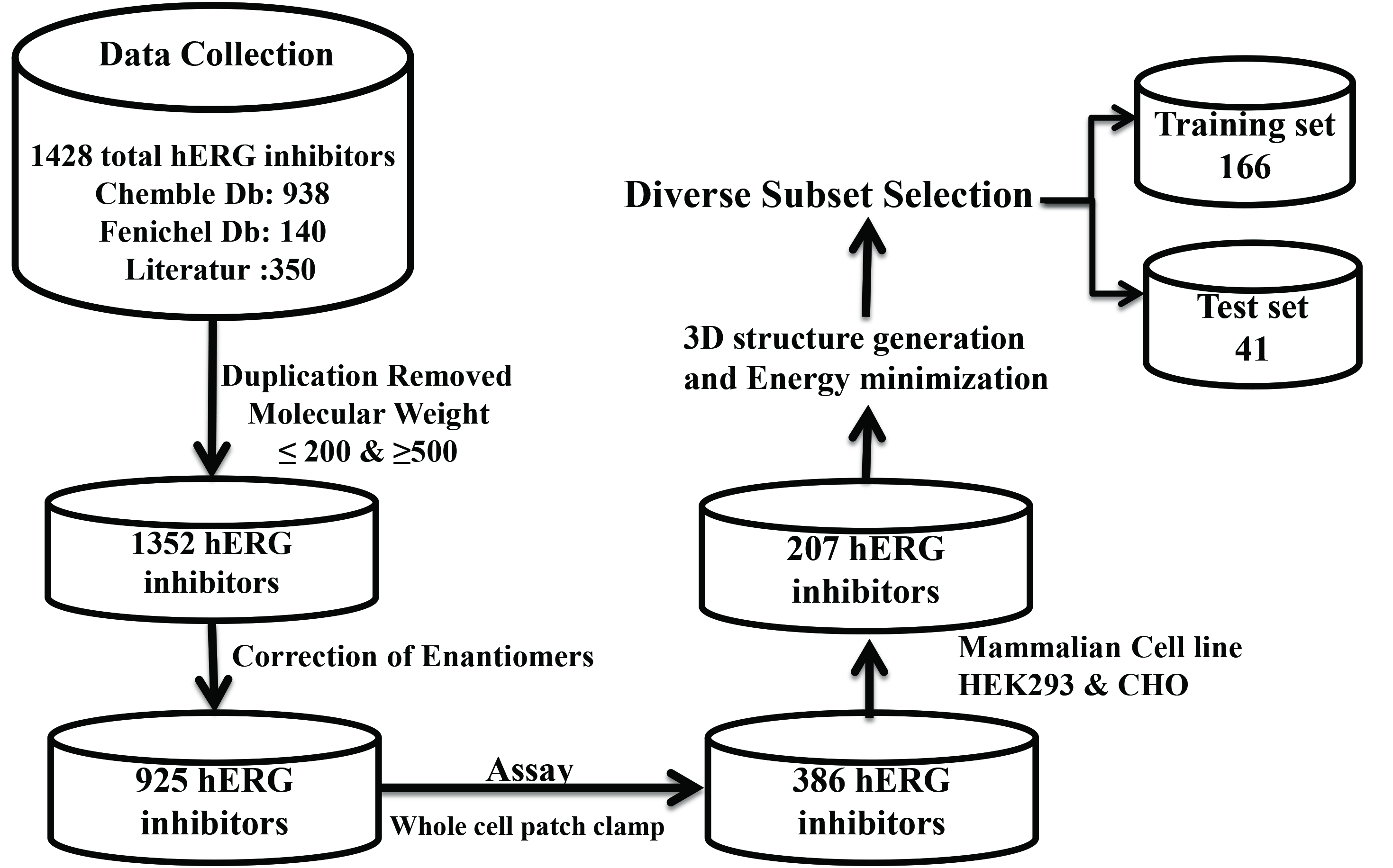


**Figure S2**. Illustrates data curation protocol showing specific filters and number of compounds obtained after applying these filters.

**Figure S3**. Correlation plot between CHO and HEK cell line studied on the whole cell patch clamp for hERG inhibition.

**Table S1.** **Comparison of pIC_50_ values of compounds taken from the literature of HEK and CHO cell line by applying whole-cell patch clamp technique.**

| **Compound Name** | **HEK pIC_50_** | **CHO pIC_50_** |
| --- | --- | --- |
| Amiodarone | 7.15 | 7.31 |
| Astemizole | 8.88 | 8.22 |
| Bepridil | 7.58 | 7.45 |
| Cisapride | 7.61 | 7.61 |
| Citalopram | 5.40 | 5.40 |
| Clarithromycin | 4.48 | 3.12 |
| Diltiazem | 4.76 | 5.13 |
| Domperidone | 7.24 | 6.79 |
| E4031 | 7.95 | 7.79 |
| Fexofenadine | 4.18 | 4.63 |
| Fluoxetine | 6.15 | 6.30 |
| Halofantrine | 7.66 | 6.70 |
| Haloperidol | 7.92 | 7.56 |
| Levofloxacin | 3.36 | 3.03 |
| Mefloquine | 5.58 | 5.25 |
| Moxifloxacin | 4.53 | 4.38 |
| Pimozide | 8.58 | 7.74 |
| Propafenone | 6.35 | 6.25 |
| Propranolol | 5.40 | 5.00 |
| Quinidine | 6.38 | 6.20 |
| Sertindole | 6.67 | 7.19 |
| Sparfloxacin | 4.47 | 4.86 |
| Terfenadine | 7.55 | 8 |
| Thioridazine | 7.09 | 6.93 |
| Tolterodine | 8.01 | 7.76 |
| Verapamil | 7.02 | 6.71 |
| Ziprasidone | 6.77 | 6.77 |

**Conformational Analysis for GRIND**

In order to obtain native, optimal and stable conformations for GRIND model various algorithms including Minimized energy, Stochastic search, docked poses and 3D standard conformations were applied to generate four independent set of conformations.

**Energy minimized conformations:** In order to generate minimum energy conformations of the training set, a built-in energy minimize the application of MOE(I 2013) was used. This application calculates atomic coordinates of the compounds at local minima of the molecular energy function. The conformations have been calculated by applying large-scale nonlinear optimization techniques(Gill, Murray et al. 1981) for which force on the atom is zero. To minimize our dataset after adding hydrogens MMFF94(Gill, Murray et al. 1981) force field based algorithm was applied for calculation of geometry constraints by using MOE’s angle, chirl, distance and dihedral restraints.

**Conformations obtained by stochastic search:** To generate stochastic search conformation MOE’s inherent fragment based methodology was applied. This technique includes initialization of bond rotation and random inversion of all chiral centers following bond rotation to randomized dihedral angles (possibly 30° and 60°). Perturbation of all atomic position/s with energy minimization of all cartesian coordinates was performed(Thai and Ecker 2009).

**Induced Fit Docking Conformation:** MOE(I 2013) was used to generate the docking conformations for the entire training set compounds within the open state of hERG homology model using induced fit docking protocol. The binding cavity was defined by reported mutagenesis residues Thr_623, Ser_624, Val_625, Ser_649, Tyr_652 and Phe_656(Lees-Miller, Duan et al. 2000, Mitcheson, Chen et al. 2000, Saxena, Zangerl-Plessl et al. 2016). Docking run was performed using placement method Alpha PMI and London dG scoring function. The best scoring pose of each ligand was selected for the further model building.

**Standard 3D conformation:** Various studies confirmed that degree of molecular freedom or extended conformations has an impact on biological activity(Gasteiger, Rudolph et al. 1990). Therefore, the standard 3D conformations of training set were generated using CORINA.direct(Gasteiger, Rudolph et al. 1990) web-based software. It handles stereochemical information of compounds to generate minimum energy conformation by neutralizing formal charges, removing salts and counter ions if any and orients the 3D structure with reference to moment of inertia. CORINA combines monocentric fragments of compounds with standard bond length, bond angles, dihedrals angle and produces minimum energy conformations of all compounds that were later used as an input in order to build GRIND model.

In order to probe ligand-protein interaction profile with the binding cavity of hERG highly potent inhibitors (Figure 1) were selected by applying hit to lead approaches. For the first time ever hit to lead approaches were used in our study for the selection of potent inhibitors in order to probe ligand protein interaction profiles.

**Template Selection Protocol**

**Ligand and Lipophilic Efficiency Profiling**: Briefly, lipophilic efficiency (LipE) distribution profiles of the data set of hERG inhibitors have been calculated using equation 1 in Excel spreadsheet as described by Freeman et al, (Freeman-Cook, Hoffman et al. 2013). The clogP values were calculated using Bio Loom software (<http://www.biobyte.com/bb/prod/bioloom.html>).

$\mathbf{LipE =}\boldsymbol{pIC}_{\boldsymbol{50}}\mathbf{- clogP}$ **Eq.1**

LipE profiles of the current data set of hERG inhibitors are shown in figure S4 A.

However, Ligand efficiency (LE) was calculated using equation 2 as described by Hopkins et al, (Hopkins, Groom et al. 2004). Where $\Delta G$ reflect the binding free energy that was calculated using equation 3 and HA represents heavy atom count.

$\mathbf{LE}=\left( \Delta\mathbf{g} \right)=-\Delta\mathbf{G}/\mathbf{HA}_{(\mathbf{non}-\mathbf{hydrogen} \mathbf{atoms})}$ **Eq.2**

Hopkins et al, described inhibitory potency values IC_50_  can be substituted for dissociation constant potency Kd (Hopkins, Groom et al. 2004). Kuntz and co-workers also confirmed this experimentally (Kuntz, Chen et al. 1999).

$\Delta\mathbf{G}=-\mathbf{RTlnKd}$ **Eq.3**

LE calculation was performed for a temperature of 310 K and given in kcal per heavy atom**.**

Overall, LE of the data set decreases with increase in molecular weight as shown in figure S4B. Therefore, a size independent Fit Quality (FQ) score (Figure S4C) was obtained by normalizing ligand efficiency with respect to ligand efficiency scale as described in equation 5. FQ scores ≥1 (equation 6) show the optimal fit of ligands within the respective binding cavity. However, FQ < 1 is indicative of suboptimal binding of ligands.

$\mathbf{LE}\_\mathbf{Scale}=\mathbf{EXP}(-\mathbf{0}.\mathbf{044}*\mathbf{HA})$ **Eq.4**

$\mathbf{FQ}=\mathbf{LigE}/\mathbf{LigEScale}$ **Eq.5**


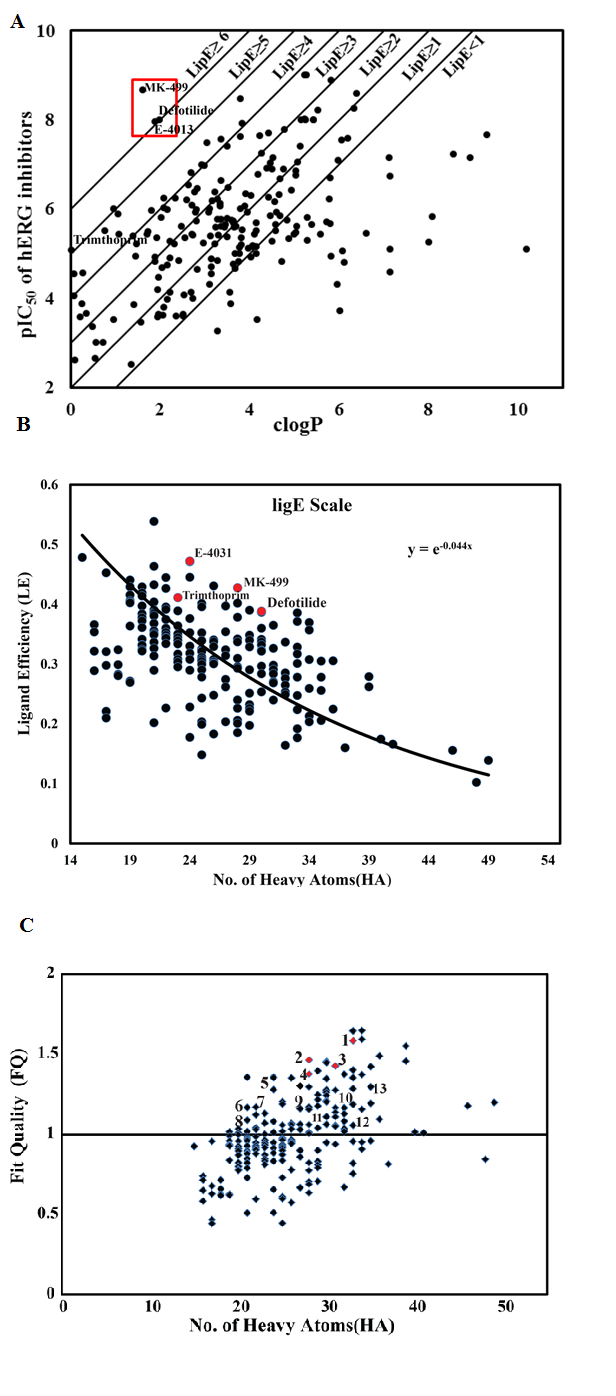


**Figure S4:** Lipophilic and Ligand Efficiency Profiles of hERG inhibitors **(A)** Plot of Potency versus clogP of hERG inhibitors. **(B)** Plot of Number of heavy atoms versus Ligand efficiency. **(C)** The graph indicates fit quality scores versus a number of heavy atoms.

**Molecular Docking Protocol**

In order to get deeper inside of potential ligand-protein interactions, molecular docking of 13 selected ligands (Figure 1) was performed using the recent cryo-EM structure of hERG in its open conformational state(Wang and MacKinnon 2017) close state homology model of hERG proposed by Stansfled et al,(Stansfeld, Gedeck et al. 2007) using software package GOLD v 5.3(Jones, Willett et al. 1997). Briefly, the binding cavity was identified by selecting an area of 18A° and 15A° from the center in open and closed conformational states of hERG homology models respectively. The selected area covered previously known mutagenesis data including aromatic (Tyr_652, Phe_565) and polar (Tyr_623, Ser_624, Val_625, Ser_649 and Gly_648) residues. These residues are located on bottom loop of pore helix and line the inner cavity, whereas, Gly_648 is present on the inner helix. 100 genetic runs per ligands in both conformational states were performed. In total 1300 poses were generated in each conformation. GOLD software ranked the pose according to GOLD score. However, to remove any biasness in the GOLD score, all poses were rescored using different scoring functions like ASE, alpha HB, affinity DG and London DG scoring functions using Molecular Operating Environment (MOE) v 2013.08. In order to build a consensus among all the scoring functions, top 10 poses of each ligand were compared on the basis of different scoring functions. Best pose of each ligand was selected on the basis of the accumulative sum of scoring values. In recent studies, this approach has been used to get most potent hERG inhibitors for feature selection in order to improve virtual screening process (Li, Meng et al. 2018, Onawole, Kolapo et al. 2018). Finally, 13 best poses from each open and closed state were obtained. These final docked poses were further used for building a common feature pharmacophore for hERG inhibitors. The complete docking and pose evaluation protocol is given in Figure S3.


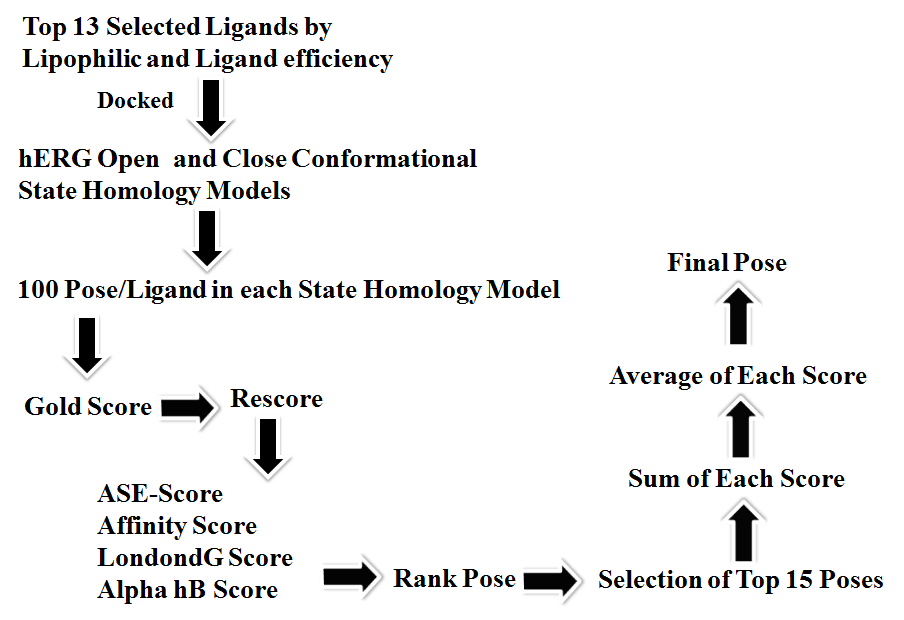


**Figure S5**. Docking and pose evaluation protocol with consensus scoring method.

**Virtual Screening Protocol**


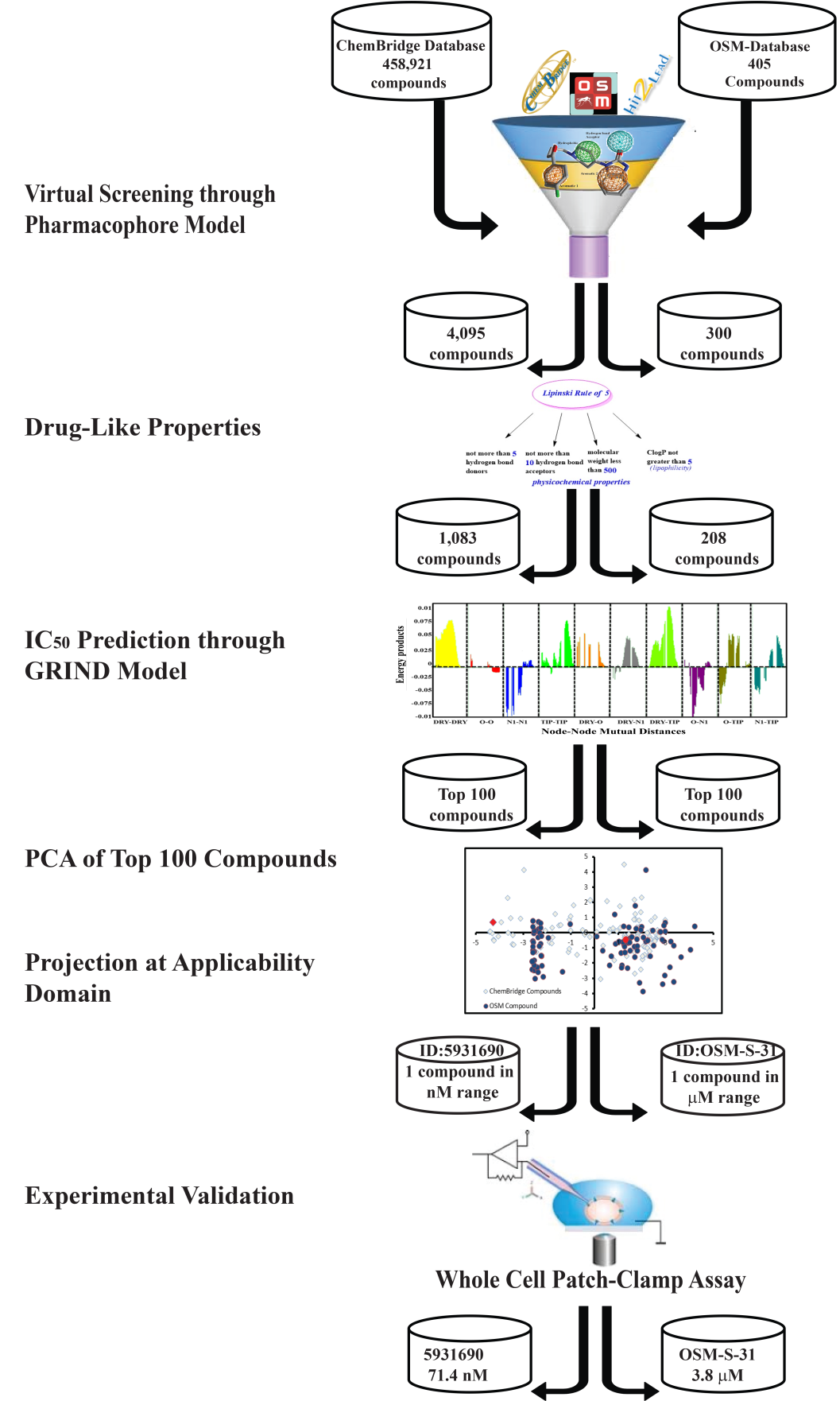


**Figure S6.** Complete hit selection protocol for experimental validation.

**Figure S7.** Showing PCA analysis, hit selection for experimental validation. White diamonds are compounds of Chembridge database and blue circle are OSM database compounds. Red points are the hits selected for experimental validation.

Applicability Domain and Similarity Assessment

According to PCA analysis, the selected compounds by virtual screening exhibit the same chemical space as of the training model. To further assess the similarity of compounds, the Tanimoto coefficient was calculated using the molecular fingerprint Molecular Accession System (MACCS) keys. The 5931690 exhibit 50% however OSM-S-31 exhibit 56% overlap with training data.

NMR Spectra of selected compounds

Db ID: 5931690:

*NMR 1H (400 MHz, DMSO-D6) δ: 2.51 (3H, s), 3.73 (3H, s), 5.40 (1H, br. s), 6.79–6.84 (2H, br. m), 6.90–6.95 (2H, br. m), 7.21–7.24 (2H, m), 7.25–7.35 (5H, m), 7.38–7.46 (3H, m), 7.66  (1H, s), 10.2 ppm  (1H, br. s)*





**Figure S8.** NMR spectra of 2-(2-[(4-methoxyphenyl) amino]-1-propen-1-yl-3, 4-diphenyl)-1, 3-thiazol-3-ium bromide. **Db ID: 5931690.**


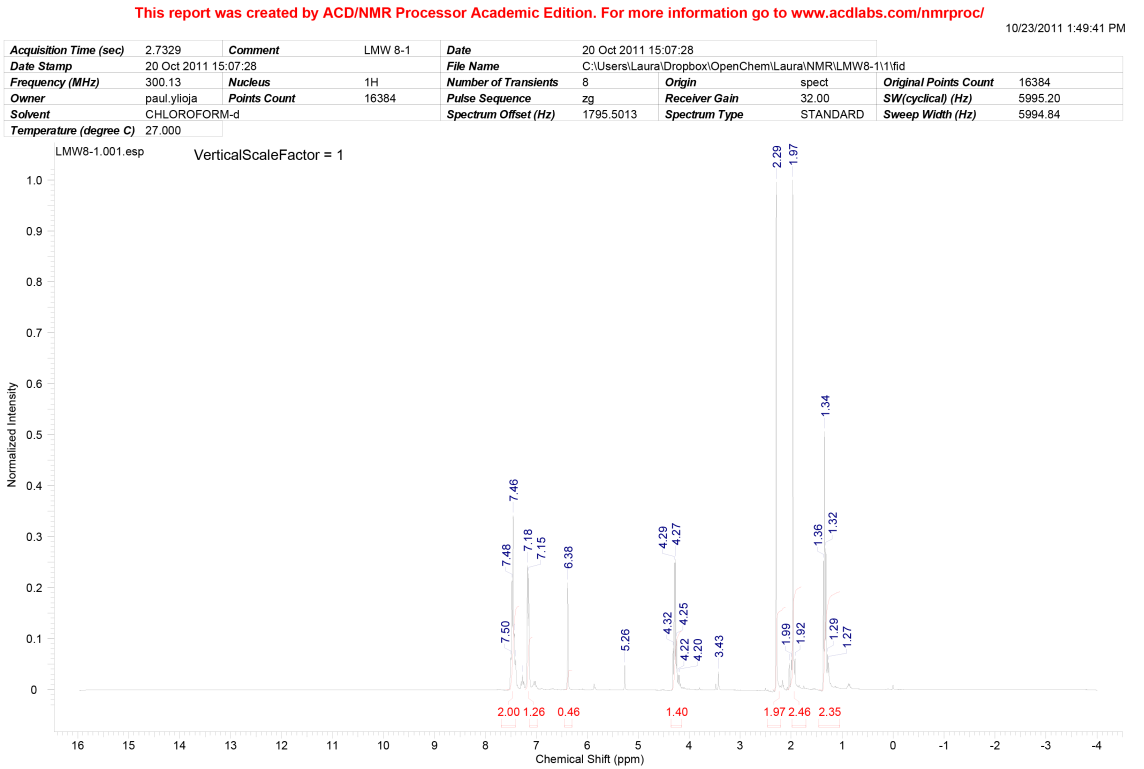


**Figure S9.** NMR spectra of Ethyl 2,5-dimethyl-1-phenyl-1H-pyrrole-3-carboxylate **Db ID: OSM-S-31.**

**Results Section**

**Principal component analysis (PCA)**

**
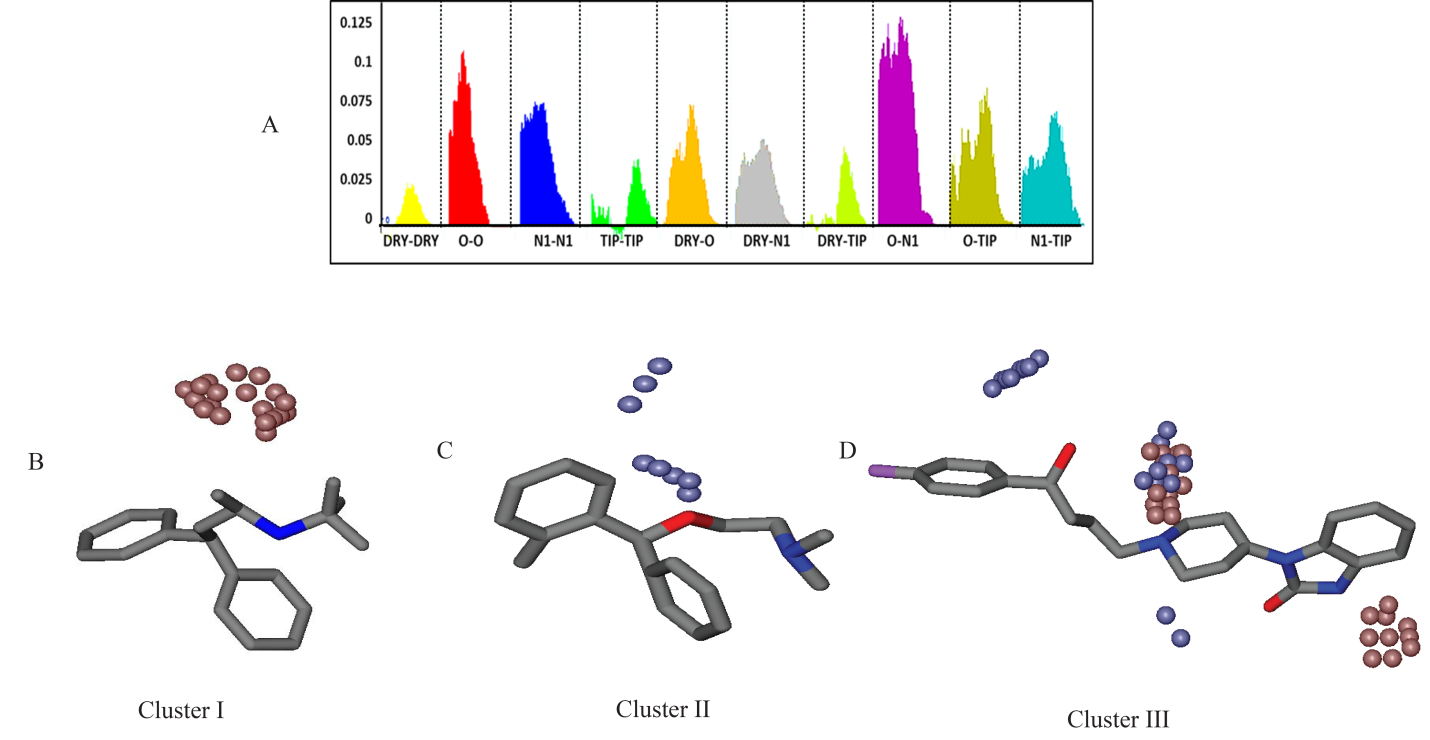
**

**Figure S10.** Representative compounds from PCA clusters and their respective virtual receptor site that defines the potential region for ligand protein interaction: A) PCA correlogram B) Cluster I compound showing the presence of single hydrogen bond donor group C) Cluster II compound showing the presence of single hydrogen acceptor group D) Cluster III compound showing the presence of multiple hydrogen acceptor and donor group.

**Molecular Docking:**

Briefly, the nitrile group in final binding solution of antiarrhythmic agent **MK499** in the open state homology model of hERG showed hydrogen bond interaction with hydroxyl group of Ser_624 and carbonyl group of Tyr_623 as shown in Figure S10A However, in close state model π-π interactions have been observed between aryl moieties at both sides of the molecules and Tyr_652 of subunit B and subunit D Figure S10B. The interaction pattern in the closed state of the channel further validates DRY-DRY correlogram in Figure 6 that depicts the distance between two hydrophobic contours surrounding highly potent hERG blockers. Previously, various studies reported the π-π interactions between aromatic moieties in hERG inhibitors and Tyr_652 or Phe_656 (Lees-Miller, Duan et al. 2000, Mitcheson, Chen et al. 2000). Additionally, the role of Ser_624 in making hydrogen bond interaction with basic nitrogen atom of the ligand has been demonstrated by various authors in the past(Stansfeld, Gedeck et al. 2007, Karczewski, Wang et al. 2009) This further strengthen the validity of selected ligand binding solutions as a templates for pharmacophore modeling in open as well as close state of hERG.


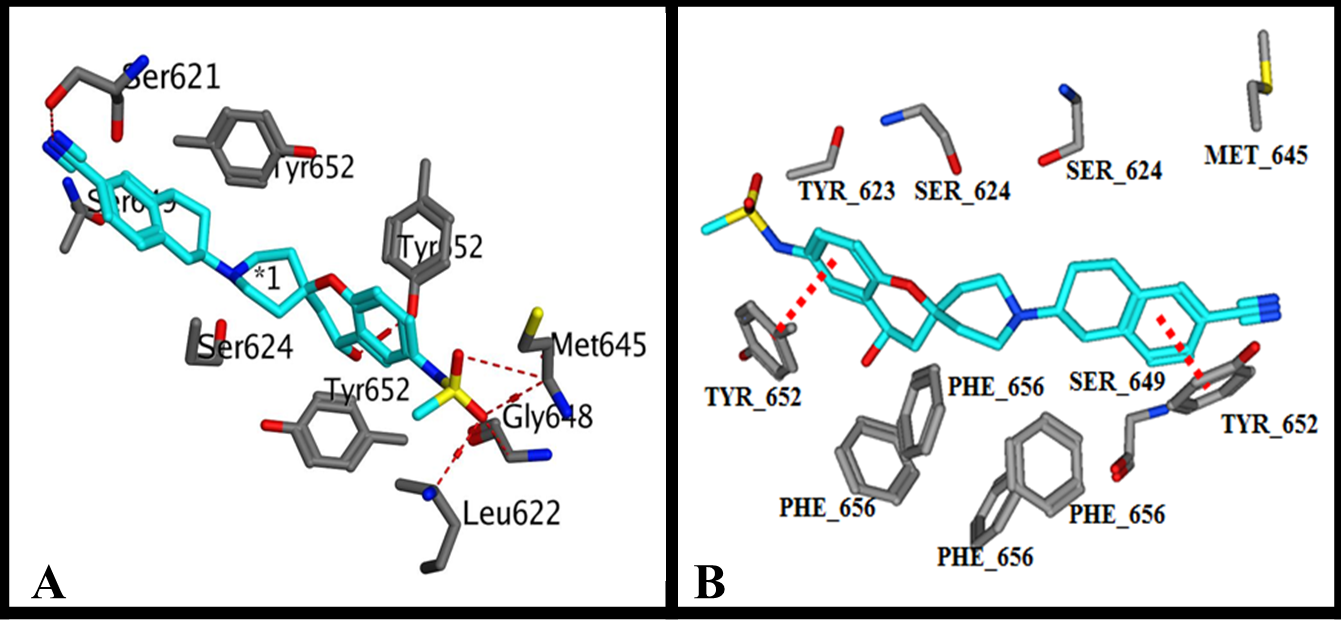


**Figure S11.** Ligand-protein interaction profile of MK499 (A) Finally selected docked pose in open conformational state of hERG homology model showing hydrogen bond interaction of MK499 with polar residues Ser621, Gly648, Leu622, and Met645. (B) Finally selected docked pose of MK499 in the close conformational state of hERG homology model showed π-π interactions with aromatic residues of basal cavity Phe_656 and Tyr_652.

Similarly**, Dofetilide**, the dopamine antagonist and **9-Hydroxy Risperidone** showed π-π interactions with Tyr_652 of C and A subunit in open state of hERG as shown in Figures S10A and Figure S10C respectively. However, the two aryl moieties in the antipsychotic agent **Haloperidol** showed π-π interactions with Phe_656 and Tyr_652 of subunit B and C respectively as shown in Figure S10E. These interactions elucidate the DRY-DRY correlogram variables in Figure 6. In closed state of hERG channel, **Dofetilide**, **9-Hydroxy Risperidone** and **Haloperidol** showed hydrogen bond interaction with Ser_649, Ser_624 and π-π interactions with TYR_652 and Phe_656 as shown in figure 10 B, D and F. These interactions correspond to N1 (hydrogen bond donor contour) and DRY (hydrophobic contours) variables in DRY-N1 correlogram. A similar interaction pattern has been observed by an antipsychotic agent **Benperidol** in both open as well as the close state of the channel as shown in Figure S12.

**
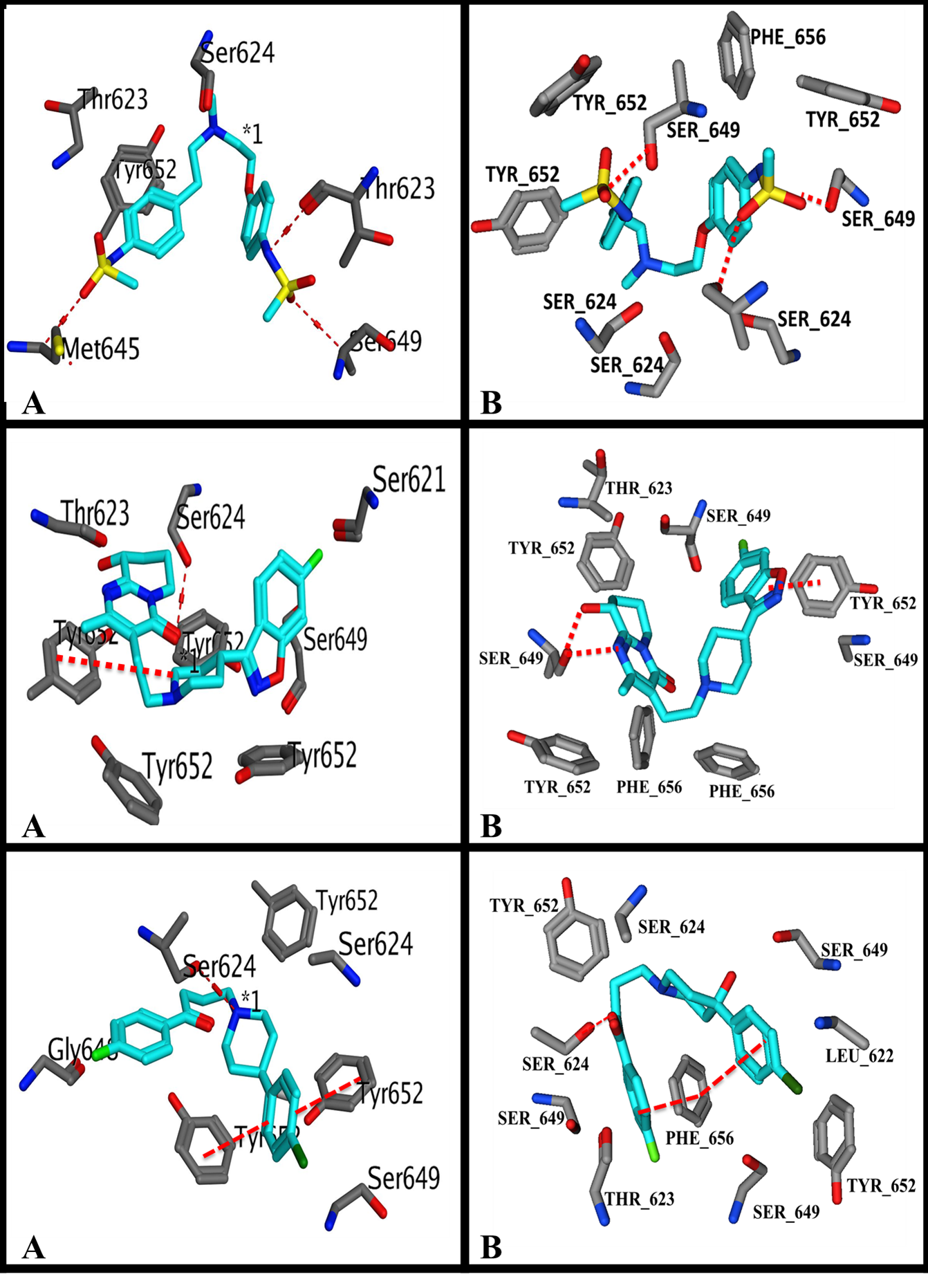
Figure S12.** Interaction profiles of Dofetilide (A, B), 9-Hydroxy Risperidone (C, D) and Haloperidol (E, F) in open and closed conformational state of hERG respectively. Respective aromatic moieties show π-π interactions with Phe656 and Tyr652 in open conformational state of hERG. In closed state of the hERG OH and C=O groups in respective compounds show hydrogen bond interaction with Ser_624 and Ser_649.

**E**

**F**

**C**

**D**

Similarly, the final docking solution of class III antiarrhythmic agent, **E4031** in open state of hERG revealed hydrogen bond interactions between the basic nitrogen of methanesulfonamide group of E4031 and the polar side chain residues, Ser624 and Ser649. Additionally, piperidine ring of the E4031 group showed hydrophobic interaction with the Tyr652 as shown in Figure S11A and Figure S11B Interestingly these interactions correspond to the hydrogen bond donor and hydrophobic features present in highly potent hERG blockers as depicted by DRY-O correlogram in Figure 6. In close state homology model of hERG, E4031 shift its position near selectivity filter and carbonyl oxygen of methanesulfonamide group form hydrogen bonding with Ser_624 as shown in figure 11B. Additionally, π-π interactions between Phe_656 and aromatic moiety of one of the aryl group of the E4031 has been identified that corresponds to GRIND mapped distance between a hydrophobic group and a hydrogen bond acceptor group within the highly potent hERG inhibitors as depicted by DRY-N1 correlogram in Figure 6. The identified interaction of E4031 within open and closed state of hERG is also in line with the previously reported interactions of E4031(Dempsey, Wright et al. 2014) that further strengthens its template conformational validity for pharmacophore modeling. A similar ligand protein interaction has been observed by all selected templates (Figure 1**)**, in open state of the hERG channel as shown in Figure S12 and Figure S13. Overall, in close state of hERG, all selected templates make more hydrogen bond interactions with the Ser_624 at selectivity filter or Ser_649 at S6 helix (Figure S12 and Figure S13)

The final pose of the antibiotic agent, **Trimethoprim** being a small structure remain intact inside the central cavity in open as well as close state of the channel. In open state homology model of hERG it showed hydrogen bond interaction between the methoxy group present at 3 and 4 positions of trimethoxyphenyl and hydroxyl group of Ser624 as shown in Figure S11C However, in close sate 3,4,5-trimethoxyphenyl is flipped and only hydrogen bonding between 4-methoxy group and Ser_624 of hERG subunit A, C, and D remain stable. Additionally, hydrogen bonding between amino group at the pyrimidine and carbonyl group of Thr_623 is formed as shown in Figure S11D.

Nevertheless, 4-Fluorobenzyl group in **Droperidol** showed π-π interactions with Tyr652 in open state of the channel. Additionally, carboxylic group of Ser_624 at hERG subunit A and D showed hydrogen bonding with amino group of benzimidazole ring and central pyridine ring respectively as shown in Figure S11E. This represents the DRY-O correlogram in Figure 6. In closed state of the channel, strong hydrogen bonding has been observed with the selectivity Ser_624 and Ser_649 as shown in Figure S12F.

**
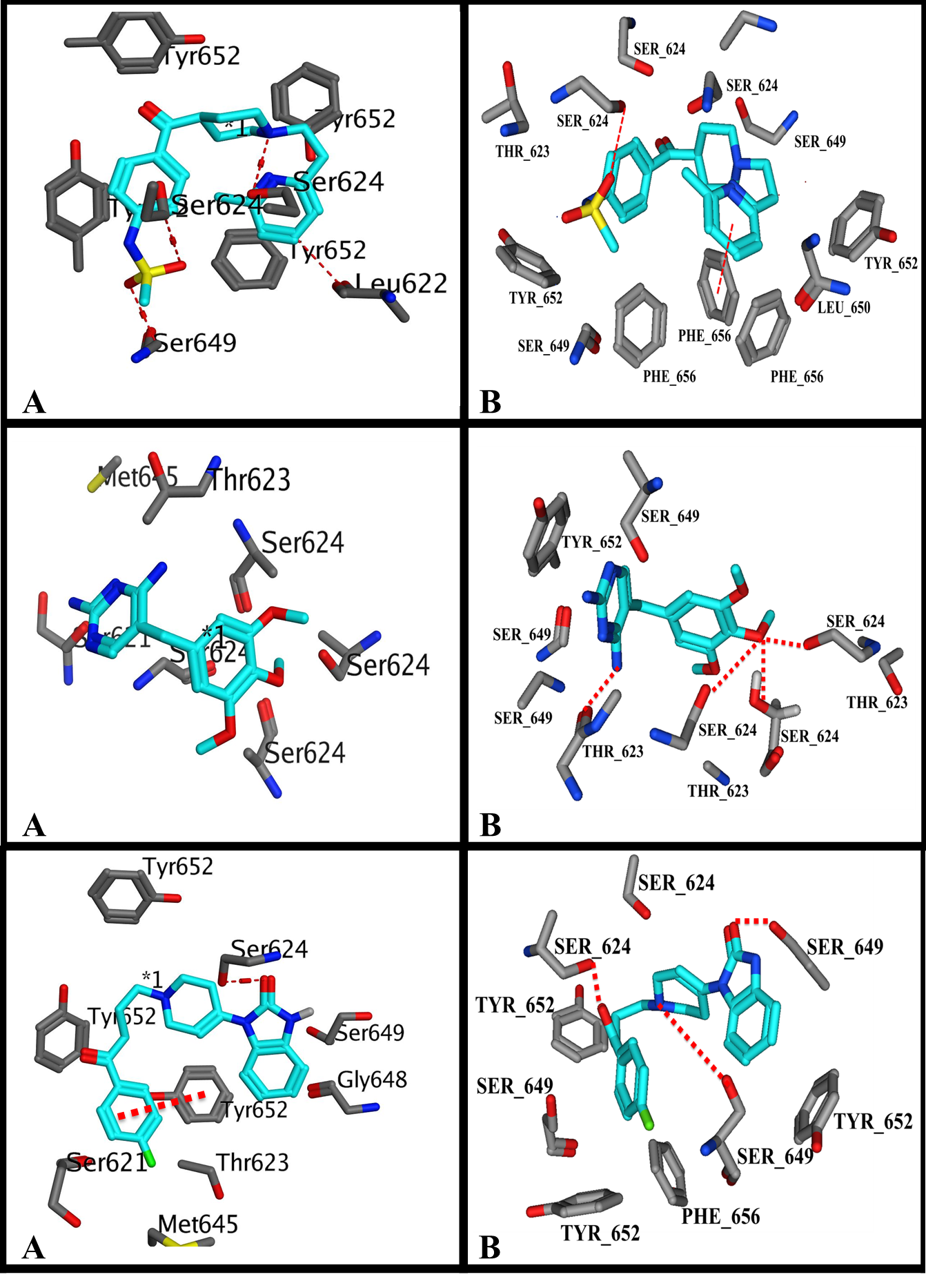
Figure S13.** Showing ligand-protein interaction profiles of (A) E4031 docked in open conformational state of hERG showing π-π interactions with Phe656 and hydrogen bonding with Ser264 (B) E4031 binding solution in close conformational state of hERG showing hydrogen bonding with Ser_624 and π-π interactions with Phe_656 (C) Final docked pose of Trimethoprim showing hydrogen bond interactions with Ser624 and Ser649 in open conformational state of hERG (D) Selected binding pose of Trimethoprim in closed conformational state of hERG homology model showing hydrogen bonding with Ser_649 with A, C and D subunits of hERG (E) Final binding pose of Droperidol showing π-π interactions with Phe656 and hydrogen bond interactions with Ser624 and Ser649 in open conformational state of hERG (F) Selected docking solution of Droperidol showing strong hydrogen bonding with Ser_624 with and Ser_649 A subunit and showing π-π interactions with Tyr_652 of closed conformational state of hERG.

**C**

**D**

**E**

**F**

**
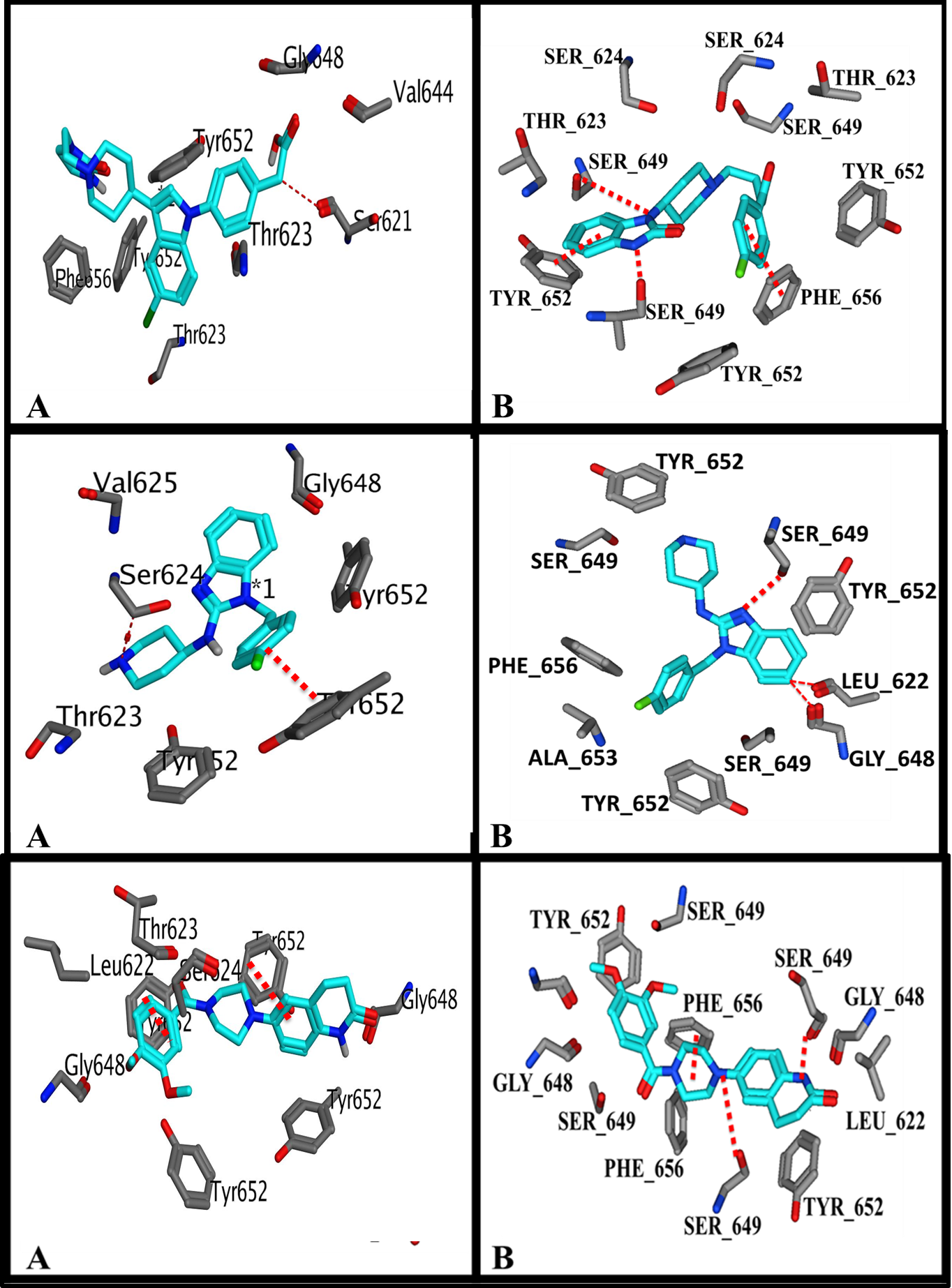
Figure S14.** Showing ligand-protein interaction profiles of (A) Benperidol docked in open conformational state of hERG showing π-π interactions with Phe656 and hydrogen bonding with Ser_621 (B) Benperidol docked in close conformational state of hERG showing hydrogen bonding with Ser649 C and B subunit and π-π interactions with Tyr_652 (C) Final docked pose of Norastemizol showing hydrogen bonding with Ser624 (D) docked pose of Norastemizol in closed conformational state of hERG model showing hydrogen bonding with Ser_649, Lue_622 and Gly_648 (E) Vesnarinone docked in open conformational state of hERG showing hydrogen bonding with Lue622 and Tyr652 (F) Final docked pose of Vesnarinone in closed conformational state of hERG model showing π-π interactions with Tyr_652 and hydrogen bonding with Ser_649.

**F**

**E**

**C**

**D**

**
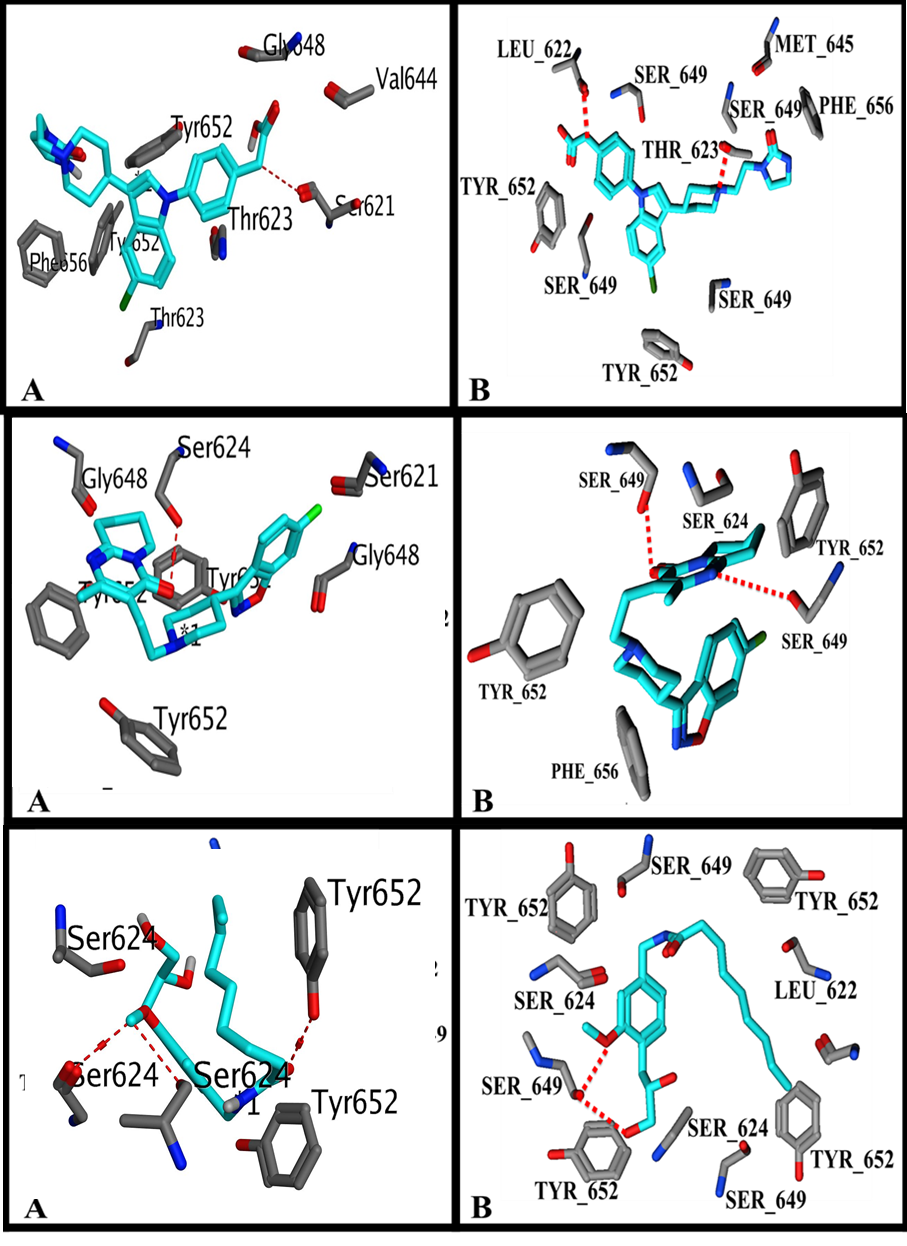
**

**G**

**F**

**E**

**C**

**Figure S15.** Showing ligand-protein interaction profiles of (A) BMLC_1835_4 docked in open conformational state of hERG showing π-π interactions with Phe656 and hydrogen bonding with Met_645 and Ser_621 (B) BMLC_1835_4 docked in close conformational state of hERG showing hydrogen bonding with Lue_622 and Thr_623 (C) Final docked pose of Risperidone showing hydrogen bonding with Ser624 and π-π interactions with Tyr652 (D) docked pose of Risperidone in closed conformational state of hERG model showing hydrogen bonding with Ser_649 B and D subunit (E) Glycerol nonivamide docked in open conformational state of hERG showing hydrogen bonding with Ser649 and Thr623 (F) Final docked pose of Glycerol nonivamide in closed conformational state of hERG model showing hydrogen bonding with Ser_624 B and C subunit of hERG channel.

**Db ID: OSM-S-31**

**Db ID: 5931690**

**References:**

Dempsey, C. E., D. Wright, C. K. Colenso, R. B. Sessions and J. C. Hancox (2014). "Assessing hERG pore models as templates for drug docking using published experimental constraints: the inactivated state in the context of drug block." Journal of chemical information and modeling **54**(2): 601-612.

Freeman-Cook, K. D., R. L. Hoffman and T. W. Johnson (2013). "Lipophilic efficiency: the most important efficiency metric in medicinal chemistry." Future medicinal chemistry **5**(2): 113-115.

Gasteiger, J., C. Rudolph and J. Sadowski (1990). "Automatic generation of 3D-atomic coordinates for organic molecules." Tetrahedron Computer Methodology **3**(6): 537-547.

Gill, P. E., W. Murray and M. H. Wright (1981). "Practical optimization."

Hopkins, A. L., C. R. Groom and A. Alex (2004). "Ligand efficiency: a useful metric for lead selection." Drug discovery today **9**(10): 430-431.

I, C. C. G. (2013). Molecular Operating Environment (MOE), Chemical Computing Group Inc. Montreal, QC.

Jones, G., P. Willett, R. C. Glen, A. R. Leach and R. Taylor (1997). "Development and validation of a genetic algorithm for flexible docking." Journal of molecular biology **267**(3): 727-748.

Karczewski, J., J. Wang, S. A. Kane, L. Kiss, K. S. Koblan, J. C. Culberson and R. H. Spencer (2009). "Analogs of MK-499 are differentially affected by a mutation in the S6 domain of the hERG K+ channel." Biochemical pharmacology **77**(10): 1602-1611.

Kuntz, I., K. Chen, K. Sharp and P. Kollman (1999). "The maximal affinity of ligands." Proceedings of the National Academy of Sciences **96**(18): 9997-10002.

Lees-Miller, J. P., Y. Duan, G. Q. Teng and H. J. Duff (2000). "Molecular determinant of high-affinity dofetilide binding toHERG1 expressed in Xenopus oocytes: involvement of S6 sites." Molecular Pharmacology **57**(2): 367-374.

Li, D.-D., X.-F. Meng, Q. Wang, P. Yu, L.-G. Zhao, Z.-P. Zhang, Z.-Z. Wang and W. Xiao (2018). "Consensus scoring model for the molecular docking study of mTOR kinase inhibitor." Journal of Molecular Graphics and Modelling **79**: 81-87.

Mitcheson, J. S., J. Chen, M. Lin, C. Culberson and M. C. Sanguinetti (2000). "A structural basis for drug-induced long QT syndrome." Proceedings of the National Academy of Sciences **97**(22): 12329-12333.

Onawole, A. T., T. U. Kolapo, K. O. Sulaiman and R. O. Adegoke (2018). "Structure based virtual screening of the Ebola virus trimeric glycoprotein using consensus scoring." Computational biology and chemistry **72**: 170-180.

Saxena, P., E.-M. Zangerl-Plessl, T. Linder, A. Windisch, A. Hohaus, E. Timin, S. Hering and A. Stary-Weinzinger (2016). "New potential binding determinant for hERG channel inhibitors." Scientific reports **6**.

Stansfeld, P. J., P. Gedeck, M. Gosling, B. Cox, J. S. Mitcheson and M. J. Sutcliffe (2007). "Drug block of the hERG potassium channel: insight from modeling." Proteins: Structure, Function, and Bioinformatics **68**(2): 568-580.

Thai, K.-M. and G. F. Ecker (2009). "Similarity-based SIBAR descriptors for classification of chemically diverse hERG blockers." Molecular diversity **13**(3): 321-336.

Wang, W. and R. MacKinnon (2017). "Cryo-EM Structure of the Open Human Ether-à-go-go-Related K+ Channel hERG." Cell **169**(3): 422-430. e410.
